# Repeated BCG treatment of mouse bladder selectively stimulates small GTPases and HLA antigens and inhibits single-spanning uroplakins

**DOI:** 10.1186/1471-2407-7-204

**Published:** 2007-11-02

**Authors:** Marcia R Saban, Helen L Hellmich, Cindy Simpson, Carole A Davis, Mark L Lang, Michael A Ihnat, Michael A O'Donnell, Xue-Ru Wu, Ricardo Saban

**Affiliations:** 1 Department of Physiology, The University Oklahoma Health Sciences Center, Oklahoma City, USA; 2 Department of Anesthesiology, University of Texas Medical Branch, Galveston, USA; 3 Department of Microbiology and Immunology The University Oklahoma Health Sciences Center, Oklahoma City, OK 73104, USA; 4 Department of Cell Biology, The University Oklahoma Health Sciences Center, Oklahoma City, OK 73104, USA; 5 Department of Urology, University of Iowa, UI Hospitals and Clinics, Iowa City, Iowa 52242-1089, USA; 6 Department of Urology, New York University, School of Medicine, New York, NY 10016, USA

## Abstract

**Background:**

Despite being a mainstay for treating superficial bladder carcinoma and a promising agent for interstitial cystitis, the precise mechanism of Bacillus Calmette-Guerin (BCG) remains poorly understood. It is particularly unclear whether BCG is capable of altering gene expression beyond its well-recognized pro-inflammatory effects and how this relates to its therapeutic efficacy. The objective of this study was to determine differentially expressed genes in the mouse bladder following repeated intravesical BCG therapy.

**Methods:**

Mice were transurethrally instilled with BCG or pyrogen-free on days 1, 7, 14, and 21. Seven days after the last instillation, urothelia along with the submucosa was removed and amplified ds-DNA was prepared from control- and BCG-treated bladder mucosa and used to generate suppression subtractive hybridization (SSH). Plasmids from control- and BCG-specific differentially expressed clones and confirmed by Virtual Northern were then purified and the inserts were sequenced and annotated. Finally, chromatin immune precipitation combined with real-time polymerase chain reaction assay (ChIP/Q-PCR) was used to validate SSH-selected transcripts.

**Results:**

Repeated intravesical BCG treatment induced an up regulation of genes associated with antigen presentation (B2M, HLA-A, HLA-DQA1, HLA-DQB2, HLA-E, HLA-G, IGHG, and IGH) and representatives of two IFNγ-induced small GTPase families: the GBPs (GBP1, GBP2, and GBP5) and the p47GTPases (IIGTP1, IIGTP2, and TGTP). Genes expressed in saline-treated bladders but down-regulated by BCG included: the single-spanning uroplakins (UPK3a and UPK2), SPRR2G, GSTM5, and RSP 19.

**Conclusion:**

Here we introduced a hypothesis-generator approach to determine key genes involved in the urothelium/sumbmucosa responses to BCG therapy. Urinary bladder responds to repeated BCG treatment by up-regulating not only antigen presentation-related genes, but also GBP and p47 small GTPases, both potentially serving to mount a resistance to the replication of the *Mycobacterium*. It will be of tremendous future interest to determine whether these immune response cascades play a role in the anti-cancer effects exerted by BCG.

## Background

Intravesical Bacillus Calmette-Guerin (BCG) has been presented as a promising option for treatment of interstitial cystitis [1]. It is even better known as the most effective agent for the treatment of high-grade superficial bladder cancer [2-4]. In this context, BCG is used to reduce both the recurrence rate of bladder tumor and to diminish the risk of its progression [2,4]. As an adjunct to transurethral resection, BCG is the treatment of choice for urothelial carcinoma in-situ (CIS) and is commonly used for recurrent or multi-focal Ta and high grade T1 bladder lesions [5,6].

It is not clear, however, how BCG alters the course of cystitis or cancer progression. One theory is that intravesical BCG corrects an aberrant immune imbalance in the bladder, leading to long-term symptomatic improvement [1].

BCG is internalized and processed by neutrophils [7,8], professional antigen-presenting cells, and urothelial tumor cells, resulting in altered gene expression and secretion of particular cytokines [9]. It was suggested that the effectiveness of BCG treatment is determined by two processes: an inflammatory one, followed by a delayed type of hypersensitivity (DTH) response [10]. Others proposed three distinct phases in the immune response to BCG. In phase 1, BCG adheres to the urothelium via interaction between the bacterial antigen 85 complex and fibronectin [6,11] in the urothelial cells. In addition to fibronectin, it has been suggested that toll-like receptors (TLRs) -2 and -4, present in immune cells, mediate BCG-induced immune responses [12-14]. Once internalized, BCG is processed both by professional antigen-presenting cells and urothelial cells, resulting in an altered gene expression [9]. This phase corresponds to the early release of so-called inflammatory cytokines (IL-1, IL-6, and IL-8 in humans) which may be responsible for certain adverse effects. Phase 2 consists of recognition of bacterial antigens by CD4 lymphocytes, which release cytokines including IL-2 and IFN-γ (TH_1 _response). This cell activation leads to phase 3, which consists of amplification of cytotoxic-populations: CD8 T cells, gamma-delta lymphocytes, macrophages, and natural killer (NK) cells. All these cells also release cytokines which then regulate the immune response to BCG [15].

IFN-γ plays an important role mediating the immune responses to BCG. Indeed, chronic administration of BCG to mice leads to an increase in IFN-γ mRNA [8] and protein [8,16], as well as genes downstream of the IFN-γ pathway [16]. The products of these genes may coordinate the cellular defense against intracellular pathogens, such as *Mycobacteria *[17,18], and therefore, influence BCG survival in diverse ways. One of these genes encodes indoleamine 2,3-dioxygenase (IDO), an enzyme that catalyses the conversion of tryptophan to N-formyl-kynurenine, which depletes tryptophan stores [16] and consequently, may starve the growth of BCG. Other IFN-γ-regulated genes include nitric inducible oxide synthase 2 (NOS2), phagocyte oxidase (phox), and natural resistance-associated macrophage protein 1 (NRAMP1) [18]. NOS2 is present in the cytosol, where it catalyses the production of nitric oxide, which traffics to the phagosome to effect pathogen killing [18]. Phox/NADPH oxidase subunits are localized to the plasma membrane, cytosol, and intracellular granular structures, and assemble on newly forming phagosome membranes. Assembled NADPH oxidase pumps electrons into the phagocytic vacuole to catalyze the conversion of O2 to superoxide and other reactive oxygen species [18]. Proton pumping by the NADPH oxidase provides charge compensation resulting in acidification of the phagosomal lumen. The combination of lower pH, chloride anion and reactive oxygen species likely result in pathogen killing [19]. NRAMP1 is also recruited to the phagosome where it catalyses cation transport and promotes phagosomal acidification [18]. Finally, the small GTPases including the guanylate-binding proteins (GBPs) and the p47 GTPases are also regulated by IFN-γ[18], are localized to the cytosol [18], and they inhibit both viral and *mycobacterium tuberculosis *replication [20].

Recently, the susceptibility to BCG was correlated with polymorphisms of the human NRAMP1 gene [21], providing interesting insights into the complexity of the genomics of BCG immunotherapy [22]. NRAMP1 is the human ortholog of *Bcg *gene [21], discovered in an animal model of orthotopic murine bladder tumors, and responsible for tumor responsiveness to BCG [23]. A single G169D mutation in the mouse *Bcg *gene sequence results in 2 distinct strains, namely *Bcgs (s-sensitive) *and *Bcgr (r-resistant)*, differing significantly in their susceptibility to early stage tuberculosis infection and their response to intravesical instillation of BCG for bladder tumor treatment [23,24].

Nevertheless, BCG's mechanism of action remains poorly understood. Although systemic reactions have been reported, a likely scenario is that exposure to BCG results in a local immune response [6]. Recently, we presented evidence that the repeated intravesical instillation of BCG into the bladder of C57Bl/6 mice induces bladder inflammation and a unique cytokine release that differs from common pro-inflammatory stimuli [8]. Here, we used the same animal model to further define the profile of bladder responses to acute (single intravesical instillation) and chronic (four weekly intravesical instillations) BCG therapy. The rationale for this design is based on clinical findings indicating that BCG therapy requires multiple dosing to achieve efficacy.

## Methods

### Animals

All animal experimentation described here was performed in conformity with the "Guiding Principles for Research Involving Animals and Human Beings (OUHSC Animal Care & Use Committee protocols #05-088 and 05-081). Ten to twelve week-old C57BL/6 female mice (The Jackson Laboratory; Bar Harbor, ME) were anesthetized, transurethrally catheterized as previously described [8], and instilled with 200 μl of one of the following substances: BCG (n = 20; TheraCys^®^-Aventis-Pasteur; total dose of 1.35 mg [19]) or pyrogen-free saline (n = 20) on days 1, 7, 14, and 21. Seven days after the last instillation, mice were euthanized with pentobarbital (200 mg/kg, i.p.), and the bladders removed, placed in RNA *later*™ (Ambion) and visualized under a dissecting microscope (Nikon SMZ 1500). As previously described [25], the urothelium along with the submucosa were separated from the detrusor muscle and used in subsequent experiments.

### Suppression subtractive hybridization (SSH)[25]

#### Construction of subtractive cDNA libraries

mRNA was isolated from total RNA using Poly(A) Quick mRNA Isolation Kit (Stratagene, La Jolla, CA) according to the manufacturer's protocol. The quality of RNA was determined by gel electrophoresis (Additional File 1).

#### cDNA synthesis and Rsa I digestion

Amplified ds cDNA was prepared from control bladder mucosa (C) and BCG-treated bladder mucosa (T) RNA using a SMART approach as described in Clontech Smart PCR cDNA Synthesis Kit User Manual, Cat# 634902 [26]. SMART Oligo II oligonucleotide and CDS primer were used for first-strand cDNA synthesis. In both cases, first-strand cDNA synthesis was started from 0.3 μg RNA in total reaction volume 10 μl. One μl of 5-times diluted first-strand cDNA was then used for PCR amplification with SMART PCR primer. 18 PCR cycles (each cycle included 95°C for 7 s; 65°C for 20 s; 72°C for 3 min) were performed. SMART-amplified cDNA samples were further digested by Rsa I endonuclease. The results of ds cDNA synthesis and Rsa I digestion are presented in Additional File 2, and primers used in the subtraction are listed in Additional File 3.

#### Subtraction procedure

Subtractive hybridization was performed using PCR-SelectTM cDNA Subtraction method in both directions (control vs. treated and treated vs. control) as described in PCR-Select cDNA Subtraction Kit User Manual, Cat#637401 [26]. Briefly, the following procedures were performed: for each direction, two tester populations were created by ligation of different suppression adaptors (Adaptors 1 and 2R). These tester populations were mixed with 30× driver excess (driver cDNA had no adaptors) in two separate tubes, denatured and allowed to renature. After the first hybridization, these two samples were mixed and hybridized together. Subtracted cDNA was then amplified by primary and secondary PCR (Additional File 4).

#### Primary PCR

25 PCR cycles with PCR primer 1 were performed for subtracted control bladder mucosa (C) cDNA and 25 cycles were performed for subtracted treated bladder mucosa (T) cDNA.

#### Secondary (nested) PCR

10 PCR cycles with Nested primers 1 and 2R were performed for both subtracted cDNA samples.

#### Construction of subtracted library

Two subtracted cDNA samples enriched with differentially expressed sequences (control bladder mucosa-specific and treated bladder mucosa-specific) obtained by secondary PCR were used for library construction. In each case, approximately 40 ng of purified cDNA was cloned into the pAtlas vector (pUC base vector) and used for *E. coli *transformation.

### Differential screening of subtracted libraries

384 (4 × 96-well plates) of randomly picked white clones from tester (T)-specific library and 96 (1 × 96-well plate) of randomly picked white clones from driver (C)-specific library were used for differential screening. All plates were grown in 100 μl of LB-amp (75 ug/ml) media for 6 hours at 37°C. 1 μl aliquots of each media were used for PCR amplification with F1S and R1S primers. After, the plates were supplied with 20% glycerol and stored at -70°C. 2.0 μl of each PCR-amplified insert (about 100 ng DNA) was arrayed in 96-well format onto duplicated nylon membranes and hybridized with P-^32^-labelled subtracted C- and subtracted T cDNA probes.

### Virtual Northern Blot Analysis

Virtual Northern blot analysis was performed to confirm differential screening results. For Virtual Northern blot analysis, SMART-amplified driver (C) and tester (T) unsubtracted cDNAs were resolved on agarose gels and transferred to Hybond-N membranes. Membranes were hybridized with P-^32^-labeled probes prepared from randomly selected differential clones found by differential screening.

### Sequencing

Plasmids from C-specific clones and T-specific clones differentially expressed and confirmed by Virtual Northern were purified and inserts were sequenced using M13dir and M13rev (for C12 clone from plate C-2, A2 from plate T-4 and for E12 clone from plate T-5) plasmid primers. Usually, M13dir primer was used, but when sequence was not of a good quality, M13rev primer was used. Sequences obtained were analyzed using the BLAST web service at NCBI [27].

### Ingenuity Pathways Analysis of BCG-specific genes

Ingenuity Pathways Analysis [IPA], (Ingenuity Systems, Mountain View, CA) is a robust and expertly curated database containing up-to-date information on over 20,000 mammalian genes and proteins, 1.4 million biological interactions, and one hundred canonical pathways incorporating over 6,000 discreet gene concepts. This information is integrated with other relevant databases such as EntrezGene and Gene Ontology [28]. IPA computes a score for each network according to the fit of the set of supplied focus genes (here, BCG-specific genes). These scores, derived from *p *values, indicate the likelihood of focus genes to belong to a network versus those obtained by chance. A score >2 indicates a ≥ 99% confidence that a focus gene network was not generated by chance alone [29]. The score for networks are based on the hypergeometric distribution calculated via the computationally efficient Fisher's Exact Test for 2 × 2 contingency tables [30]. The significance value associated with Functions and Pathways is a measure for how likely it is that genes from the dataset file participate in that function. The significance is expressed as a p-value, which is calculated using the right-tailed Fisher's Exact Test. In this method, the *p*-value is calculated by comparing the number of user-specified genes of interest (i.e. Functional Analysis Genes) that participate in a given function or pathway, relative to the total number of occurrences of these genes in all functional/pathway annotations stored in the Ingenuity Pathways Knowledge Base. In the right-tailed Fisher's Exact Test, only over-represented functional/pathway annotations, annotations which have more Functions/Canonical Pathways Analysis Genes than expected by chance ('right-tailed' annotations), are used. Under-represented annotations ('left-tailed' annotations) which have significantly fewer Functions/Canonical Pathways Analysis Genes than expected by chance are not shown. The score is not an indication of the quality or significance of the network; it simply calculates the approximate "fit" between each network and the Network Eligible Genes from the input dataset, and allows us to rank the networks accordingly [30].

### Chromatin immunoprecipitation (ChIP) quantitative real-time polymerase chain reaction (Q-PCR)-Based Assays

To confirm whether intravesical BCG treatment would alter bladder gene expression, we used chromatin immunoprecipitation (ChIP) combined with Q-PCR, as described earlier [8,31,32]. For this purpose, female C57BL/6J mice were anesthetized and instilled with 200 μl of one of the following substances: BCG (TheraCys^®^-Aventis-Pasteur; total dose of 1.35 mg) or pyrogen-free saline on days 1, 7, 14, and 21, as described above. Mice were euthanized with pentobarbital (200 mg/kg, i.p.) 24 hours after a single instillation (**BCG acute**) or 7 days after 4 weekly instillations (**Control **and **BCG repeated**). A total of 60 mice were used (20 mice per group). Bladders were removed rapidly, frozen, and were shipped to Genpathway [33] for querying the chromatin for gene transcription (Genpathway's TranscriptionPath Query assay) [34].

The urinary bladders were exposed briefly to formaldehyde for cross-linking of the proteins and DNA together, followed by sonication to fragment the DNA into pieces of approximately 300–500 base pairs. An antibody against RNA polymerase II (Abcam) was then used to precipitate the DNA transcriptome [8]. The Ab-protein-DNA complexes were purified using beads coupled to protein A. The DNA was isolated from the complexes using a combination of heat to reverse cross-linking, RNase and proteases, and then purified using phenol extraction and EtOH precipitation. The final ChIP DNAs were then used as templates in quantitative PCR reactions using primer pairs specific for each gene of interest. Quantitative PCR was carried out using Taq polymerase (iQ SYBR Green Supermix, Bio-Rad). Primer pairs were designed using Primer 3 [35]. Details of the primer sequences are given in Additional File 5. The designed primers shared 100% homology with the target sequence, but no significant homology with other sequences.

Q-PCRs were run in triplicate and the values were transferred into copy numbers of DNA using a standard curve of genomic DNA with known copy numbers. The resulting transcription values for each gene were also normalized for primer pair amplification efficiency using the Q-PCR values obtained with Input DNA (unprecipitated genomic DNA). Results are presented as Transcription Events Detected Per 1000 Cells for each gene tested.

### Statistical Analysis of ChIP/Q-PCR

The difference between two mean values was analyzed with an unpaired Student's *t*-test (GraphPad Prism software version 4.0; GraphPad Software, Inc. San Diego, CA). A nominal *p *value less than 0.05 was considered statistically significant.

## Results

### Differential screening

Differential screening yielded 14 control-specific and 41 BCG-treated-specific clones, for a total of 55 clones that were putatively differentially expressed (Additional Files 6, 7, 8, 9, 10, 11, 12). These clones were taken as preliminary results, which constituted the basis for further differential analysis. The working assumption was that some of these clones may be false positives. Therefore, a differential screening of all 55 subtracted libraries was performed and differentially expressed clones were selected for Virtual Northern analysis.

### Virtual Northern

Virtual Northern blot was performed in 14 differential clones obtained from the subtracted library enriched for control-specific sequences (*Plate C-1*: E8, A12, C7, B10, C11 and *Plate C-2*: A9, A12, B4, B6, B12, C12, E8, G8, H8) and 41 clones from the subtracted library enriched for BCG-specific sequences (*Plate T-1*: C1, D8, B10, E8, G7; *Plate T-2*: A11, B2, C10, D5, D11, F3, F5, G1; *Plate T-3*: A6, B4, B10, C9, D2, D6, D11, E1, E5, F5, G2, H3; *Plate T-4*: A2, B3, B9, C7, D4, D12, F10, G9, G11, H9; and *Plate T-5*: A3, B8, E9, E12, F11, H5). Virtual Northern blot analysis of the 14 putative differential clones obtained from the subtracted library enriched for C-specific sequences confirmed that all 14 are truly differentially expressed (Additional File 13). In other words, Additional File 13 shows that, in all 14 instances, when the un-subtracted DNA from the driver (C) and tester (T) samples were probed with the putatively differential clones, there was a greater abundance in C DNA than in T DNA. Hence, it produced a more intense hybridization signal in the C lane. As all the putative C-specific clones were confirmed to be differentially expressed, they were all subsequently sequenced.

In contrast, out of the 41 putative BCG-specific clones assayed using Virtual Northern (Additional File 14), only 32 were confirmed. In this case, to be considered a true differentially expressed clone, the T lane in Additional File 14 should exhibit a more intense hybridization signal than the C lane (opposite to the previously mentioned C-subtracted library). By examining the images for G1, H3, D11, E1, B9, C7, F10, G9 and A3, it is noticed that the confirmation criterion has not been satisfied. For example, for the C7 clone, the C lane shows a more intense hybridization signal than the T lane – the exact opposite of what would have been the case had it been a true positive. As 9 of the putatively T-specific clones (G1, H3, D11, E1, B9, C7, F10, G9 and A3) were found to be false positives, they were not analyzed further.

### Control and BCG-specific transcripts

Out of 14 control- and 32 BCG-confirmed clones, several repeats were found. Only uniquely expressed clones are presented here. Additional File 15 compiles genes that were expressed in the subtraction between BCG and control tissues and represent those genes up-regulated by BCG. Additional File 15 is divided into two parts: known and unknown genes. Additional File 16 contains genes that were selectively expressed in the control group and were down-regulated by BCG treatment.

### Ingenuity Pathway Analysis

**Figure 1:**
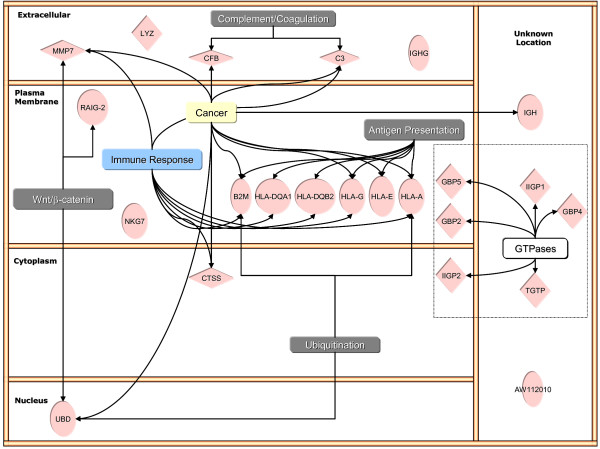
Ingenuity Pathway Analysis of BCG-induced up-regulation of bladder genes.

**Figure 2:**
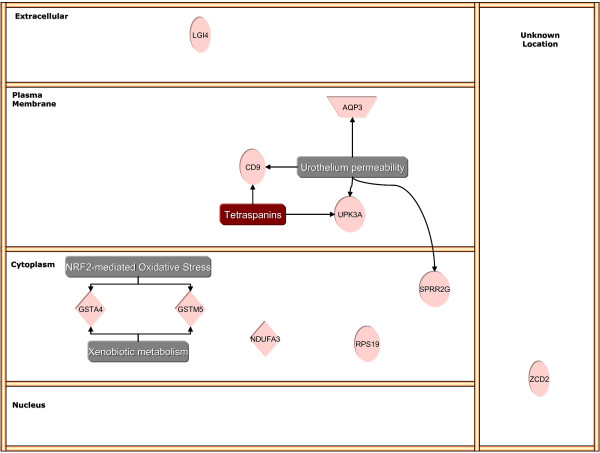
Ingenuity Pathway Analysis of BCG-induced down-regulation of bladder genes.

Figures 1 and 2 are the result of Ingenuity Pathway analysis of BCG- and control-specific transcripts annotated by cellular compartments and overlaid with significant canonical pathways, functions, and diseases. BCG-specific genes overlay several canonical pathways (complement/coagulation cascade; Wnt/β-catenin; antigen presentation), functions (immune response), and diseases (cancer), Figure 2. The dotted box in Figure 1 highlights a major group of genes encoding small GTPases (GBP2, GBP4, IIGP2, IIGP1, TGTP, and IQCK). For sake of clarity, when redundant functions were found, such as immune response and inflammation, only one function is represented. The complete IP analysis of BCG- and control-specific genes is presented in Additional Files 17 and 18.

### ChIP/Q-PCR

**Figure 3:**
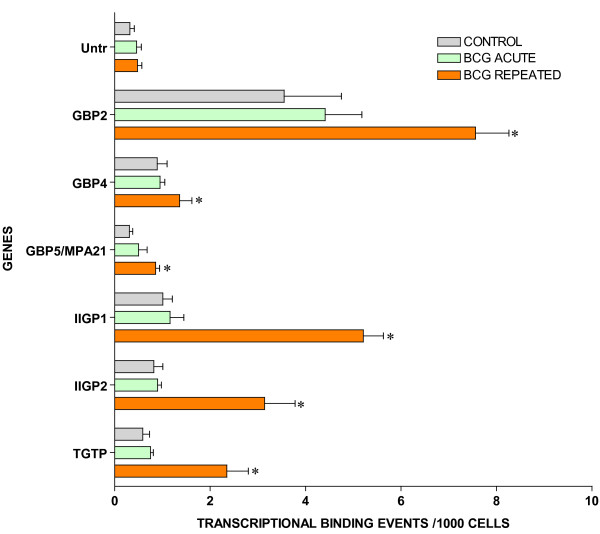
**Target validation of small GTPases by ChIP/Q-PCR**. Female C57BL/6J mice (n = 20 per group) were instilled with 200 μl of one of the following substances: BCG (total dose of 1.35 mg) or pyrogen-free saline on days 1, 7, 14, and 21, as described above. Mice were euthanized 24 hours after a single instillation (**BCG acute**) or 7 days after 4 weekly instillations (**Control **and **BCG chronic**). Bladders were exposed briefly to formaldehyde for cross-linking of the proteins and DNA together, followed by sonication to fragment the DNA. An antibody against RNA polymerase II (Abcam) was then used to precipitate the DNA transcriptome that was isolated and then purified. The final ChIP DNAs were then used as templates for Q-PCR reactions using primer pairs specific for each gene of interest (Additional File 5). Q-PCRs were run in triplicate and the averaged Ct values were transferred into copy numbers of DNA using a standard curve of genomic DNA with known copy numbers. The resulting transcription values for each gene were also normalized for primer pair amplification efficiency using the Q-PCR values obtained with input DNA (un-precipitated genomic DNA). Results are presented as "transcription events detected per 1000 cells" for each gene tested. Error bars correspond to standard deviations from the triplicate Q-PCR reactions. Control represents an untranscribed region of the genome. Asterisks indicate a statistical significant increase (p < 0.05) between BCG-treated and control and a pound sign indicates a statistical significant decrease (p < 0.05) between BCG-treated and control.

**Figure 4:**
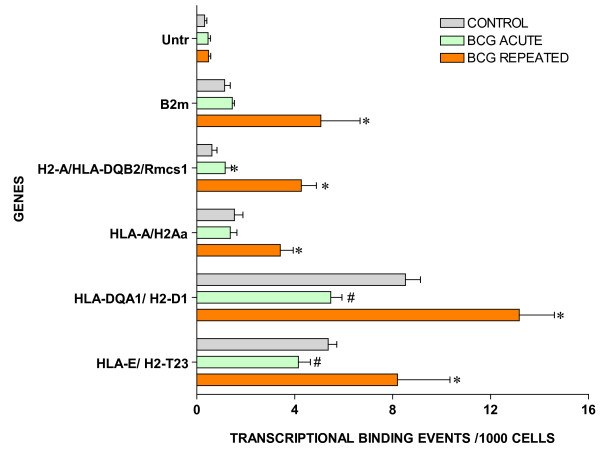
Target validation of antigen presentation related genes by ChIP/Q-PCR (see figure 3 legend).

**Figure 5:**
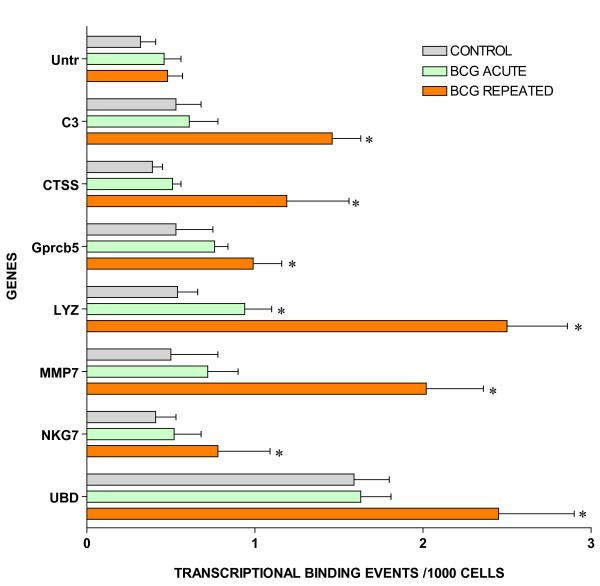
Target validation of additional BCG-induced genes by ChIP/Q-PCR (see figure 3 legend).

**Figure 6:**
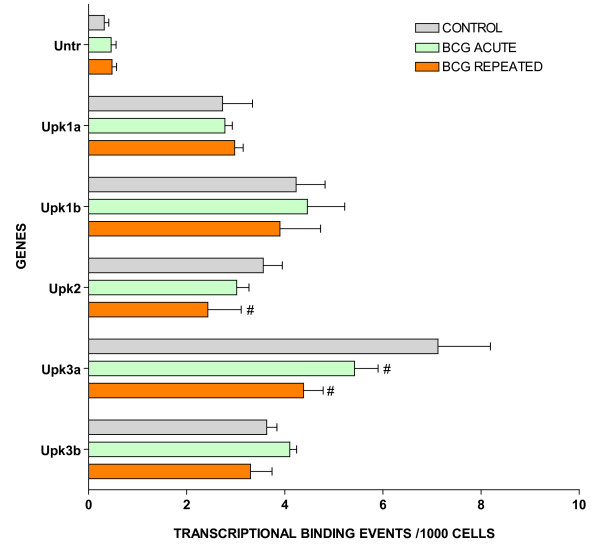
ChIP/Q-PCR of uroplakin genes in control and BCG-treated bladder mucosa (see figure 3 legend).

**Figure 7:**
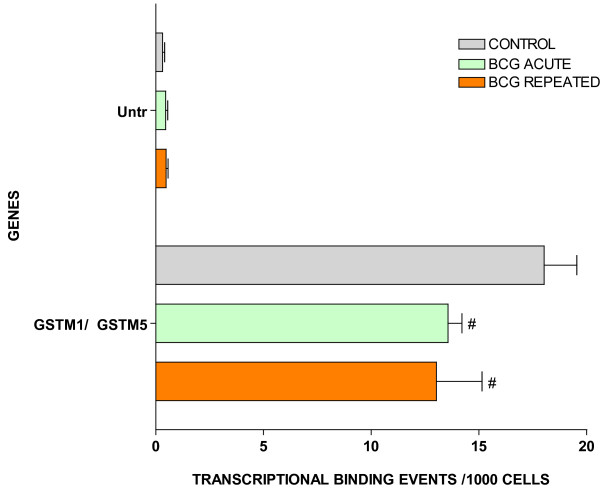
ChIP/Q-PCR of GSTM1/GSTM5 in control and BCG-treated bladder mucosa (see figure 3 legend).

For ChIP/Q-PCR, three additional experimental groups were generated using additional C57BL/6 mice that received repeated intravesical instillations of saline (control) or BCG and one group that receive acute BCG instillations. The whole mouse bladder (n = 20) was used for extraction of the chromatin (ChIP) and an antibody against RNA polymerase II (Abcam) was then used to precipitate the DNA transcriptome [8]. Q-PCR was performed in triplicate to confirm BCG-specific and control-specific genes using primers pairs described in Additional File 5 and compared to the results of an untranscribed region of the DNA. The results are being presented in groups. The first group is presented in Figure 3 and corresponds to GTPase-related transcripts. It is apparent that all GTPase related transcripts were up-regulated in response to repeated BCG treatment but not by acute BCG. The next group corresponds to genes related to antigen presentation (Figure 4). It can be noted that all antigen presentation-related genes were up-regulated by repeated BCG treatment while some genes, such as HLA-DQA1 and HLA-E, were down-regulated by acute BCG treatment (Figure 4). All other Q-PCR results for BCG-specific genes are present in Figure 5 and indicate that all tested genes were up-regulated by repeated but not acute BCG treatment. Regarding control-specific genes, we tested not only UPK3a that was found to be differently expressed in the subtraction of control minus BCG, but also several other members of this family. All members of the uroplakin family tested were significantly expressed in the control mouse bladder when compared to the untranscribed region, whereas repeated BCG treatment down-regulated UPK2 and UPK3a (Figure 6). Another control specific gene (GSTM1) was also confirmed to be down-regulated by both acute and repeated BCG treatment (Figure 7).

**Figure 8:**
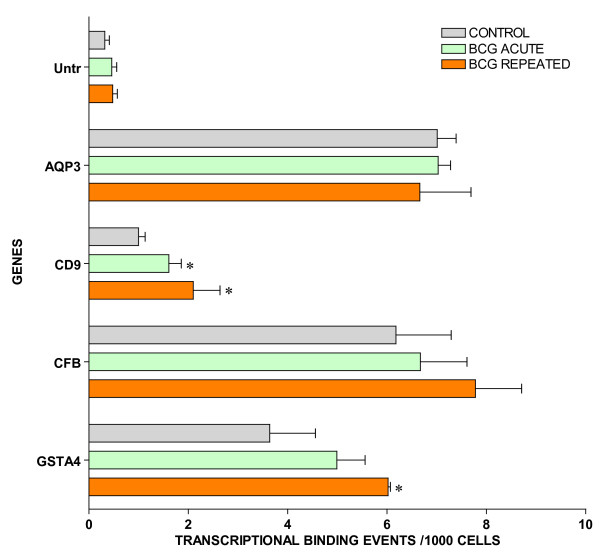
ChIP/Q-PCR of SSH-selected genes (see figure 3 legend).

Out of twenty SSH-selected transcripts, sixteen were confirmed by ChIP-Q-PCR, and four were not altered or presented an inverse response (Figure 8). This is the case of AQP3 that was found to be a control-specific gene (Figure 2) and CFB a BCG-specific gene (Figure 1) that did not present alterations in Q-PCR values. In addition, GSTA4 and CD9 that were expected to be down-regulated by BCG treatment (Figure 2), presented increased Q-PCR results in response to this treatment (Figure 8).

## Discussion

We used a highly effective method for differential gene analysis, termed suppression subtractive hybridization (SSH), which has been developed for the generation of subtracted cDNA libraries. It is based primarily on suppression PCR and combines normalization and subtraction in a single procedure [36]. The normalization step equalizes the abundance of cDNAs within the target population and the subtraction step excludes the common sequences between the target and driver populations. In a model system, the SSH technique enriched for rare sequences over 1,000-fold in one round of subtractive hybridization [36]. Unlike microarrays, which mainly identify moderate to high abundant genes, leaving for the investigator to set the criteria for differential expression, SSH identifies clones that are differentially expressed at very low levels and subtracts all the transcripts commonly expressed in both samples. It is possible that some of the extremely low-level gene expression is not biologically significant, as it might arise from 'random transcription' [37]. Therefore, we confirmed the differential expression of twenty eight SSH-selected transcripts by *QRT-PCR *and the results were highly correlated.

The rationale for a single time point for SSH, i.e. repeated BCG instillation was to keep the number of variables within a reasonable limit. We understand that using 4 weeks-instillations only may not identify preceding or proceeding events as we indicated previously [38-40]. Therefore, the results of the regulatory network presented here should be viewed as a snapshot of the bladder transcriptome at the point of maximal chronic inflammatory response [8]. In attempt to expand our findings, we used two time points for the Q-PCR, acute and chronic BCG instillation. Nevertheless, we understand that this is limited. In this context, the value of hypothesis generator research is to point out to putative pathways involved in this therapy. However, genes identified as important can now be followed and their function assessed over time by other techniques.

Another limitation of the present methodology is that the mucosal layer contains the urothelial layer and the lamina propria which involves other cell types (fibroblasts, myofibroblasts, etc.) in addition to urothelial cells that may underlie the transcriptomes identified.

In search of a possible pathway that would explain, at least in part, the properties of BCG as the gold standard treatment for bladder cancer, we used a combination of SSH, differential display, and Virtual Northern and discovered three major groups of genes: members of the small GTPase signaling molecules; those involved in antigen presentation (HLAs); and genes down-regulated by BCG, including uroplakin 3a and uroplakin 2. These groups of genes seem to be a specific bladder response to BCG therapy since they were not represented in the control- and LPS-treated SSHs [25].

Next, we used a combination of ChIP assay and Q-PCR to validate SSH-selected genes. ChIP/Q-PCR has the advantage of using a quantitative PCR method to assess genes of interest in the bladder transcriptome. The ChIP/Q-PCR assay only measures genes that are actively transcribed, in contrast to cDNA array technologies that query RNA accumulation. The disadvantage of the ChIP/Q-PCR method is the amount of chromatin necessary for ChIP, which limits this analysis to the whole bladder and not to the specific mucosal layer that was used to construct the SSH. Nevertheless, 80% of the SSH-selected transcripts were validated by ChIP/Q-PCR and this confirmation success rates compares favorably with other gene expression studies. Both SSH and ChIP/Q-PCR results have to be taken in the context that migrating inflammatory cells [8], in addition to resident cells, contributed to the measured values. Because our ChIP/Q-PCR results were obtained with whole bladders, it is not clear whether any single network may be operative in a particular cell type. However, this approach can potentially identify previously unrecognized connections among pathways and, therefore, suggests new hypotheses for the mechanistic action of BCG.

It seems that both time and dose of BCG are of essence. Most of the BCG-induced genes were validated by ChIP/Q-PCR in bladders that received repeated BCG treatment but, with few exceptions, not in the acute group. One of the possible explanations for the need of repeated treatment is based on the findings that BCG replicates poorly and survives within phagosomes [41]. Thus, multiple doses of BCG are necessary to achieve full effect. In addition, it has been suggested that poor replication of BCG may facilitate chronicity by limited generation of Ag-derived peptides, which in turn leads to reduced antigen presentation by HLA molecules, and causes a substantial delay in the development of acquired immune responses [41]. Indeed, CD8+ T cells that differentiate during infection of mice with BCG undergo only limited activation during the first 7 days of infection. The response peaks during the third week of infection, followed by a protracted and reduced contraction phase [41]. Evidence exists that the delay in generating a rapid primary CD8+ T cell is due to the reduced generation of antigenic load *in vivo*. In addition, infection of mice with a higher dose of BCG results in rapid priming of CD8+ T cell responses, which occurs 1–2 weeks earlier than with the low dose, and is followed by increased contraction [41]. Thus, for pathogens that display poor *in vivo *growth (such as BCG), the dose of the pathogen can enormously modulate the differentiation of CD8+ T cell response. The main BCG-induced pathways discovered here are involved in host adaptive immune response (HLA) and host mediated pathogen destruction (GTPases). These findings add an additional layer of complexity to the time- and concentration-dependent responses to BCG.

### IFN-γ – induced GTPases

We have previously shown that repeated BCG instillation in the mouse bladder leads to a strong cytokine response, including a significant increase in the production of IFN-γ and IL-17 proteins and their mRNAs [8]. IL-17 is an important cytokine not only in the early neutrophil-mediated inflammatory response, but also in T cell-mediated IFN-γ productionand granuloma formation in response to pulmonary infection by BCG [42]. The urinary bladder cells respond to interferons secreted during infection by the transcriptional up-regulation of as many as a thousand genes [43]. This remarkable transition prepares cells and organisms for resistance to infection, and many IFN-regulated gene products are players in well-understood resistance programs. Strangely, however, many of the most abundantly induced proteins are GTPases whose functions are not well understood [18,43]. The present results indicate that BCG induces up-regulation of several of the small GTPase molecules. At least two of such families are represented in the bladder responses to BCG: the GBPs (GBP1, GBP2, and GBP5[44]) and the p47GTPases (IIGTP1, IIGTP2, TGTP). The Mx family of proteins was not represented in the BCG SSH, but this group of proteins is induced mainly by IFN-α/β, rather than IFN-γ [45]. Others have described that the GBPs are the most abundant proteins that accumulate in response to IFN-γ stimulation [18]. Indeed, suppression-subtractive hybridization differential libraries from IFN-γ-stimulated primary mouse embryonic fibroblasts and, from a mouse macrophage cell line, in each case with reference to unstimulated cells, resulted in >35% of the total clones sequenced as representatives of GTPases (65-kDa and 47-kDa families) [46].

The association of p47 GTPases with membranous compartments such as the endoplasmic reticulum and the Golgi, implicate these molecules in intracellular membrane trafficking or processing [47]. The products of these genes are known to coordinate cellular defense against intracellular pathogens such as *Mycobacteria *[17,18], possibly by promoting acidification of the phagosome [48] and elimination of the intracellular pathogen [49]. The recently identified p47 GTPases are one of the most effective cell-autonomous resistance systems known against intracellular pathogens in the mouse [18,48], and have been shown to be essential for immune control *in vivo *of *Listeria monocytogenes, Toxoplasma gondii*, and *Mycobacterium tuberculosis *(*Mtb*) [20]. In addition, mice lacking LRG-47 failed to control *Mtb *replication [20]. This defective bacterial killing in IFN-γ-activated LRG-47^-/- ^macrophages was associated with impaired maturation of *Mtb*-containing phagosome vesicles that otherwise recruited LRG-47 in wild-type cells. Thus, LRG-47 may serve as a critical vacuolar trafficking component used to dispose of intracellular pathogens like *Mtb *[20]. Therefore, it is expected that this group of GTPases may limit the activity and survival of BCG in the mouse bladder. In addition to limiting BCG activity, this group of GTPases is part of the inflammatory response. In particular, human GBP-1 and GBP-2 modulate the endothelial cell responses to inflammatory cytokines [50,51]. The finding that GBP-1 expression is inhibited by potent activators of endothelial cell proliferation such as VEGF and bFGF [52], leads to the hypothesis that GTPases are in the cross-road of inflammation and vascular proliferation. More recently, it has been proposed that GBPs can regulate the anti-angiogenic responses of endothelial cells to inflammatory cytokines [50]. Together, these results indicate that GTPases can limit the viability of BCG and influence its vascular response. To make sense of these findings, we searched for human homologues of GTPases. The human homologues for the GBP family have been described whereas human homologues of the p47 GTPases have not been cloned, although searches of the human genome indicate that they exist [47]. Therefore, there are no reports of p47 GTPases function in humans [53]. Taken together, these results suggest that in the case of BCG vaccine, these GTPases may act as a controlled-release mechanism for the agent infecting the bladder.

### Antigen presentation (B2M, HLA-A, HLA-DQA1, HLA-DQB2, HLA-E, HLA-G, IGHG, and IGH)

Another major group of BCG-up-regulated genes encompasses both class I heavy chain (HLA-A, HLA-E, and HLA-G) and class II alpha chain (HLA-DQA1 and HLA-DQB2) paralogues of human lymphocyte antigens (HLA). The class I molecule is a heterodimer consisting of a heavy chain and a light chain (beta-2 microglobulin [B2M]) and is expressed by all nucleated cells. Class I molecules play a central role in the immune system mainly by presenting peptides to class I-restricted CD8+ T cells. Class I-binding peptides are typically derived from the endoplasmic reticulum lumen but also include 'cross-presented' exogenous antigens [54]. The class II molecule is a heterodimer consisting of an alpha (DQA) and a beta chain (DQB), both anchored in the membrane. It plays a central role in the immune system by presenting peptides derived from exogenously acquired proteins antigens to class II-restricted CD4+ T cells. Class II molecules are expressed by 'professional' antigen presenting cells (APC: B Lymphocytes, dendritic cells, macrophages). HLA class I plays a well recognized role in tumor recognition and rejection but important roles for class II have also been indicated in recent years. In particular, this includes 'priming' of cytotoxic class I-restricted T cells in a class II-restricted T cell-dependent manner [55,56]. Total or selective losses of HLA class I antigens (classified into seven HLA class I altered phenotypes) represent one of the main routes of tumor escape from immune surveillance in several malignancies including bladder cancer [57-60]. A down-regulation of HLA-ABC and beta 2-microglobulin have been reported in microdissected tumor tissue derived from bladder carcinomas [61]. This mechanism may represent a major factor for the down-regulation of HLA class I expression and in the subsequent direct recognition of cancer cells by cytolytic T lymphocytes [61]. This regulatory mechanism is frequently reversible by IFN-γ treatment [61] and contributes significantly to the therapeutic effect of BCG immunotherapy for bladder cancer [62]. Therefore, up-regulation of antigen presentation genes in response to repeated BCG administration is likely contributory to and perhaps necessary for the immune-mediated cytotoxicity of this agent, as well as IFNγ-mediated effects. The BCG-derived stimulus for HLA up-regulation could include pathogen-associated molecular patterns (PAMPS) otherwise known as Toll-Like Receptor (TLR) agonists which drive dendritic cell maturation and therefore HLA up-regulation [63]. Further investigation of these issues in the context of acute versus chronic BCG infection in the bladder is warranted.

### Uroplakins

Our results also show, for the first time, that BCG treatment selectively alters the expression of some of the uroplakins. Uroplakins are a group of integral membrane proteins found exclusively in differentiated mammalian urothelial cells [64-66]. There are four major uroplakins, UPIa, UPIb, II and UPIIIa and one minor uroplakin, UPIIIb. As the major protein constituents of the apical membrane plaques that cover almost the entire urothelial surface, these proteins play an indispensable role in maintaining the bladder's permeability barrier function. Consistent with this notion, recent experimental work demonstrated that the loss of UPII or UPIIIa in the knockout mice increased the urothelial permeability to water and urea. Damaged urothelial barrier has been associated with non-infectious cystitis, although whether uroplakin loss is involved in such a process remains unknown. Interestingly, some of the uroplakins, particularly UPIa, can serve as the urothelial receptor for type 1-fimbriated E. coli, acting to anchor the bacteria and as the point of entry into the urothelial cells [67-70]. In our current study, we found that UPK2 and UPK3a, two single membrane spanins, but not UPK1a and UPK1b, two tetraspanins, are down-regulated in chronically treated bladders by BCG. This is highly unexpected because all UPKs were previously believed to be expressed coordinately, meaning that they are invariably expressed simultaneously under physiological conditions. Whether down-regulation of UPKs represents a benefit to repeated BCG infection or a host response to reduce infection is currently unknown, but will be an interesting subject to pursue in the future.

## Conclusion

We used the subtraction technique in an attempt to find alterations in the host urothelium and chronically infiltrating host immune cells by subtracting out the control urothelium messages. Thus, we expected to find changes related to (chronic) host immunity, host-pathogen relationships and alterations in the mucosa. Our results indicate that BCG induces both host adaptive immune response (HLA) and host mediated pathogen destruction (GTPases) with a down-regulation of uroplakins. The GTPases appear to be dedicated to the BCG response, since with the exception of GBP2, their resting levels in normal urothelium are negligible (Figure 3). Most of the GTPase genes are downstream of IFN-γ that has been used as an adjuvant treatment for bladder cancer [71-75]. It will be interesting to determine whether the discovered molecules could be a target for increased BCG efficacy or whether the molecules themselves have any anti-tumor activity. It will also be interesting to find out whether some of the uroplakins can serve as the urothelial receptor(s) for BCG and whether their down-regulation reflects an increased internalization of the bacteria-receptor complex from the cell surface into the urothelial cells.

## Competing interests

The author(s) declare that they have no competing interests.

## Authors' contributions

MRS participated in its design, carried out the animal experiments, removed the tissues, extracted the RNA, performed suppression subtractive hybridizations, and performed sequence alignments; HLH participated in the design of the study regarding SSHs and target validation; CS treated the animals with BCG; CAD was responsible for the animal husbandry; MLL consulted RS regarding HLA, NADPH, and helped drafting the manuscript; MAI participated in the experimental design and helped drafting this manuscript; MAO consulted RS regarding clinical implications of BCG treatment, data interpretation, and helped drafting the manuscript; W-RW consulted RS regarding uroplakins, data interpretation, and helped drafting the manuscript. RS conceived of the study, developed annotation and *in silico *analysis, and drafted the manuscript.

## Pre-publication history

The pre-publication history for this paper can be accessed here:



## Supplementary Material

Additional file 1 Agarose gel electrophoresis of RNA samples. C = saline-treated and T=BCG-treated. Click here for file

Additional file 2 Agarose gel electrophoresis of ds cDNA synthesis and *Rsa I *digestion. Lane 1-SMART-amplified C cDNA (driver); Lane 2-SMART-amplified T cDNA (tester); Lane 3-*Rsa I *digested C cDNA; and Lane 4-*Rsa I *digested T cDNA M = 1 kb DNA size markers. Click here for file

Additional file 3 Table 1 Oligonucleotides used Click here for file

Additional file 4 Agarose gel electrophoresis of primary and secondary PCR products. Lane M = 1 kb DNA size markers, Lane 1 = primary PCR of C subtracted cDNA; Lane 2 = primary PCR of T subtracted cDNA; Lane 3 = secondary PCR of C subtracted cDNA; Lane 4 = secondary PCR of T subtracted cDNA; Lane 5 = unsubtracted C cDNA; and Lane 6 = unsubtracted T cDNA. Click here for file

Additional file 5 Table 2. QOCR Primers Click here for file

Additional file 6 Differential screening of plate C-1 from C (driver) subtracted library was subjected to differential screening using driver-specific (A) and tester-specific (B) subtracted probes. Click here for file

Additional file 7 Differential screening of plate C-2 from C (driver) subtracted library was subjected to differential screening using driver-specific (A) and tester-specific (B) subtracted probes. Click here for file

Additional file 8 Differential screening of plate T-1 from T (tester) subtracted library was subjected to differential screening using driver-specific (A) and tester-specific (B) subtracted probes. Click here for file

Additional file 9 Differential screening of plate T-2 from T (tester) subtracted library was subjected to differential screening using driver-specific (A) and tester-specific (B) subtracted probes. Click here for file

Additional file 10 Differential screening of plate T-3 from T (tester) subtracted library was subjected to differential screening using driver-specific (A) and tester-specific (B) subtracted probes. Click here for file

Additional file 11 Differential screening of plate T-4 from T (tester) subtracted library was subjected to differential screening using driver-specific (A) and tester-specific (B) subtracted probes. Click here for file

Additional file 12 Differential screening of plate T-5 from T (tester) subtracted library was subjected to differential screening using driver-specific (A) and tester-specific (B) subtracted probes. Click here for file

Additional file 13 Virtual Northern blot analysis of differential clones obtained from control bladder (C) subtracted library. A = Plate C-1 and B = Plate C-2 Click here for file

Additional file 14 Virtual Northern blot analysis of differential clones obtained from BCG-treated bladder (T) subtracted library. A = Plate T-1; B = Plate T-2; C = Plate T-3; D = Plate T-4; and E = Plate T-5. Click here for file

Additional file 15 Table 3. Genes up-regulated by BCG Click here for file

Additional file 16 Table 4. Genes down-regulated by BCG. Click here for file

Additional file 17 Table 5. Ingenuity Summary BCG. Click here for file

Additional file 18 Table 6. Ingenuity Summary Control. Click here for file
