# Supplementary figures and images for: Repeated BCG treatment of mouse bladder selectively stimulates small GTPases and HLA antigens and inhibits single-spanning uroplakins
Source: BMC Cancer. 2007 Nov 2;7:204. doi: 10.1186/1471-2407-7-204 (PMC2212656; doi:10.1186/1471-2407-7-204)

M

C

T

3 kb

2 kb

1,5 kb

1 kb

0,75 kb

0,5 kb

0,25 kb

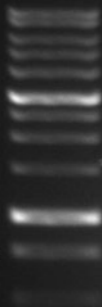

Supplement: Additional file 1 — Agarose gel electrophoresis of RNA samples. C = saline-treated and T=BCG-treated. [file 1471-2407-7-204-S1.pdf]

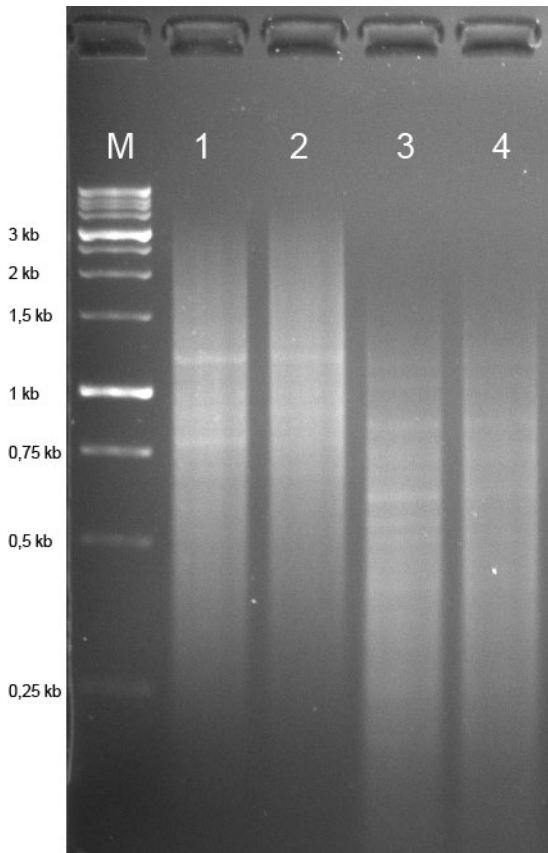

Supplement: Additional file 2 — Agarose gel electrophoresis of ds cDNA synthesis and Rsa I digestion. Lane 1-SMART-amplified C cDNA (driver); Lane 2-SMART-amplified T cDNA (tester); Lane 3-Rsa I digested C cDNA; and Lane 4-Rsa I digested T cDNA M = 1 kb DNA size markers. [file 1471-2407-7-204-S2.pdf]

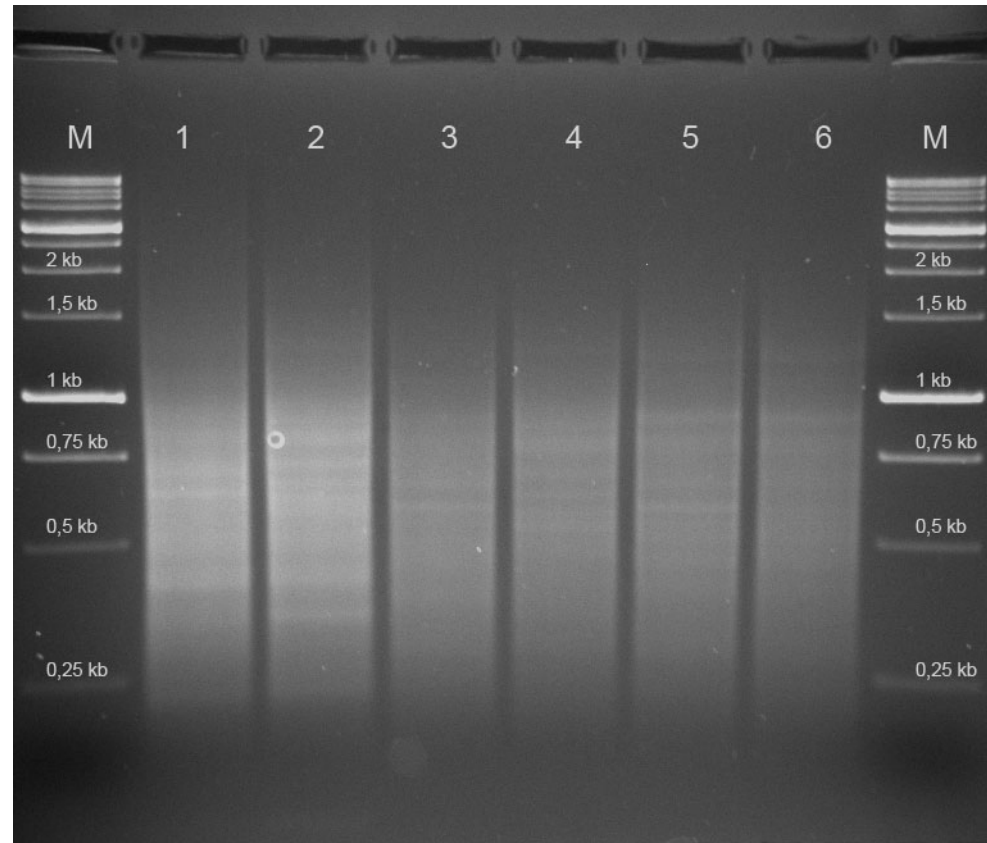

Supplement: Additional file 4 — Agarose gel electrophoresis of primary and secondary PCR products. Lane M = 1 kb DNA size markers, Lane 1 = primary PCR of C subtracted cDNA; Lane 2 = primary PCR of T subtracted cDNA; Lane 3 = secondary PCR of C subtracted cDNA; Lane 4 = secondary PCR of T subtracted cDNA; Lane 5 = unsubtracted C cDNA; and Lane 6 = unsubtracted T cDNA. [file 1471-2407-7-204-S4.pdf]

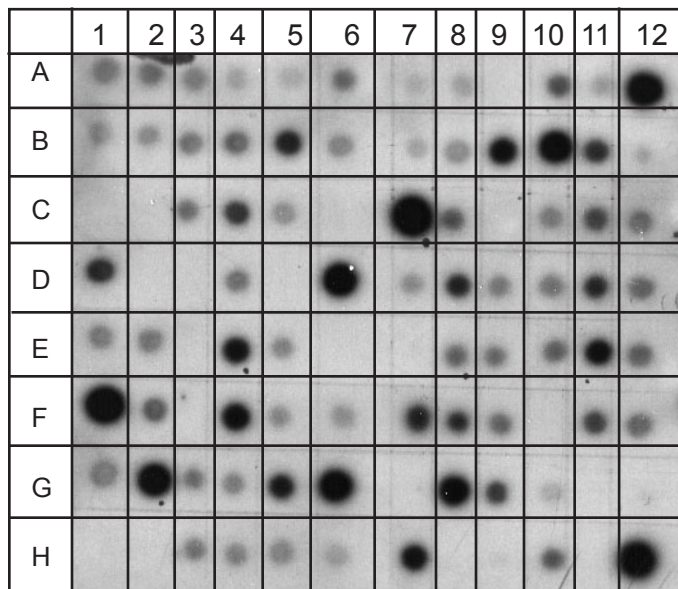

A = DRIVER-SPECIFIC

PLATE C-1

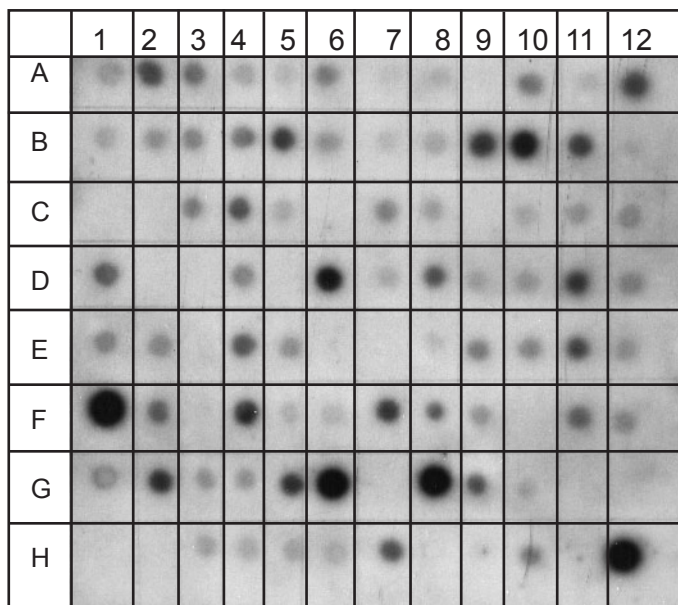

B = TESTER-SPECIFIC

Supplement: Additional file 6 — Differential screening of plate C-1 from C (driver) subtracted library was subjected to differential screening using driver-specific (A) and tester-specific (B) subtracted probes. [file 1471-2407-7-204-S6.pdf]

PLATE C-2

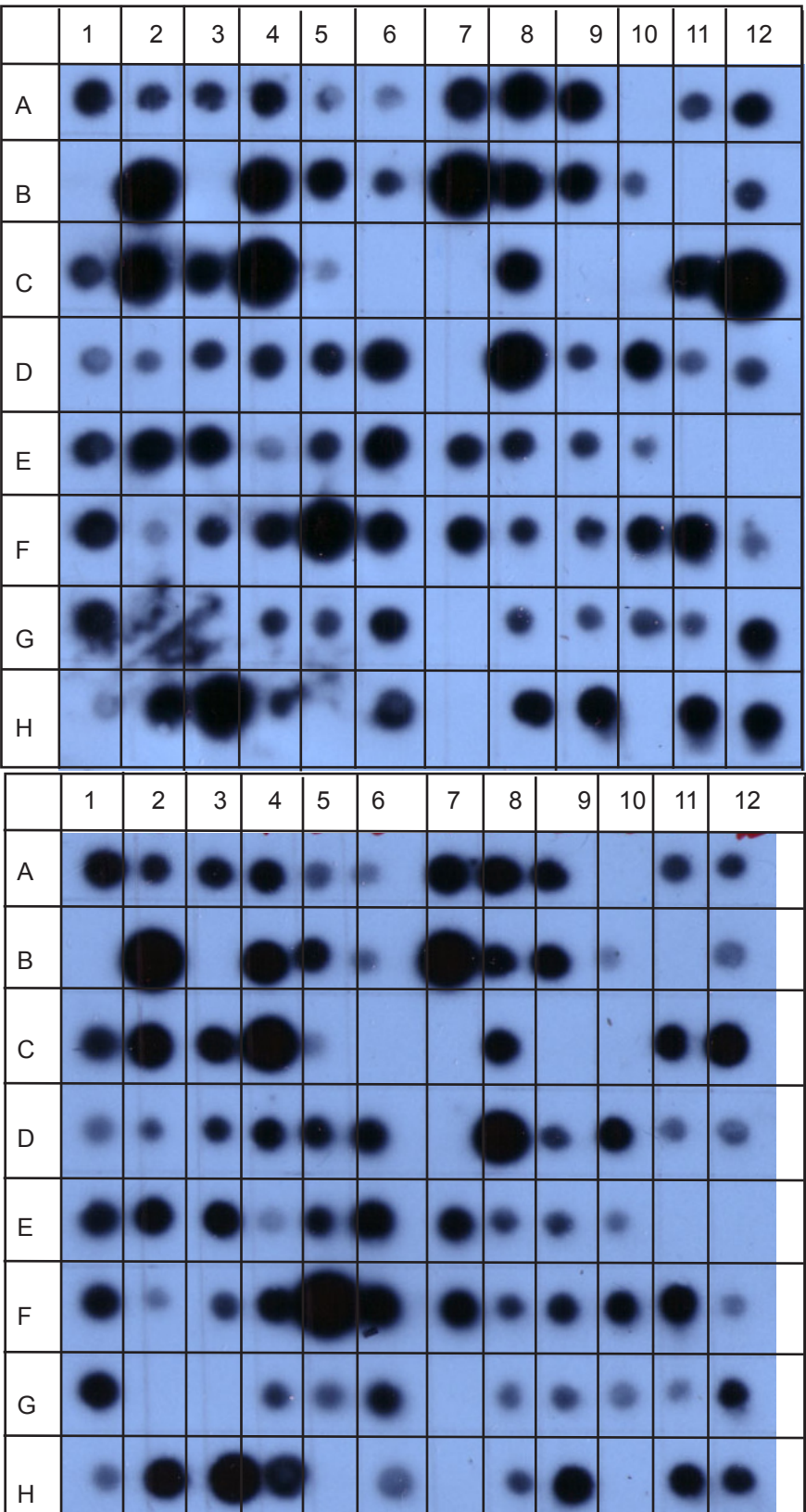

A = DRIVER-SPECIFIC

B = TESTER-SPECIFIC

Supplement: Additional file 7 — Differential screening of plate C-2 from C (driver) subtracted library was subjected to differential screening using driver-specific (A) and tester-specific (B) subtracted probes. [file 1471-2407-7-204-S7.pdf]

PLATE T-2

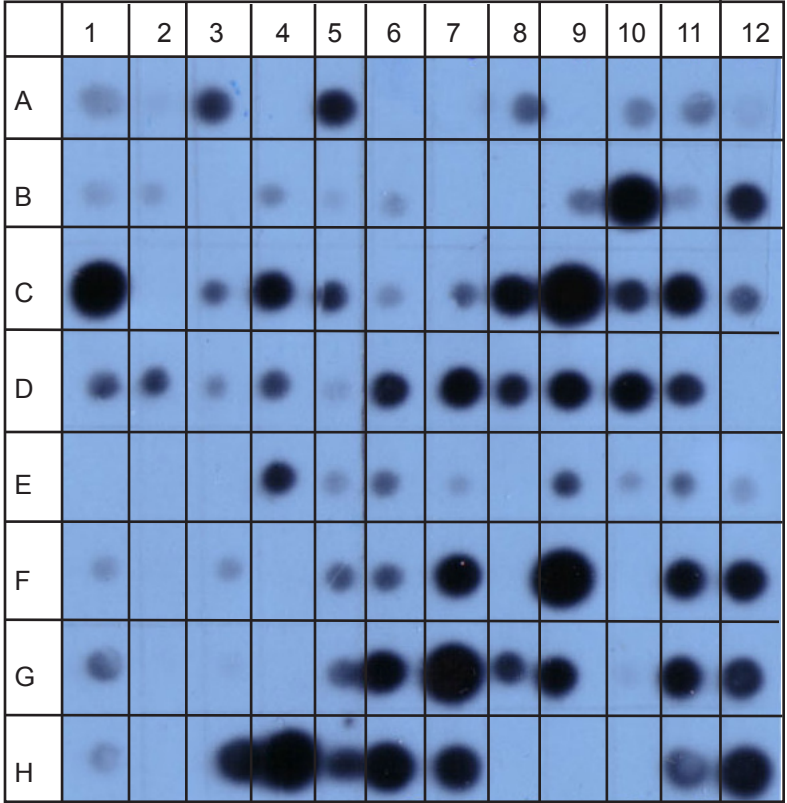

A = DRIVER-SPECIFIC

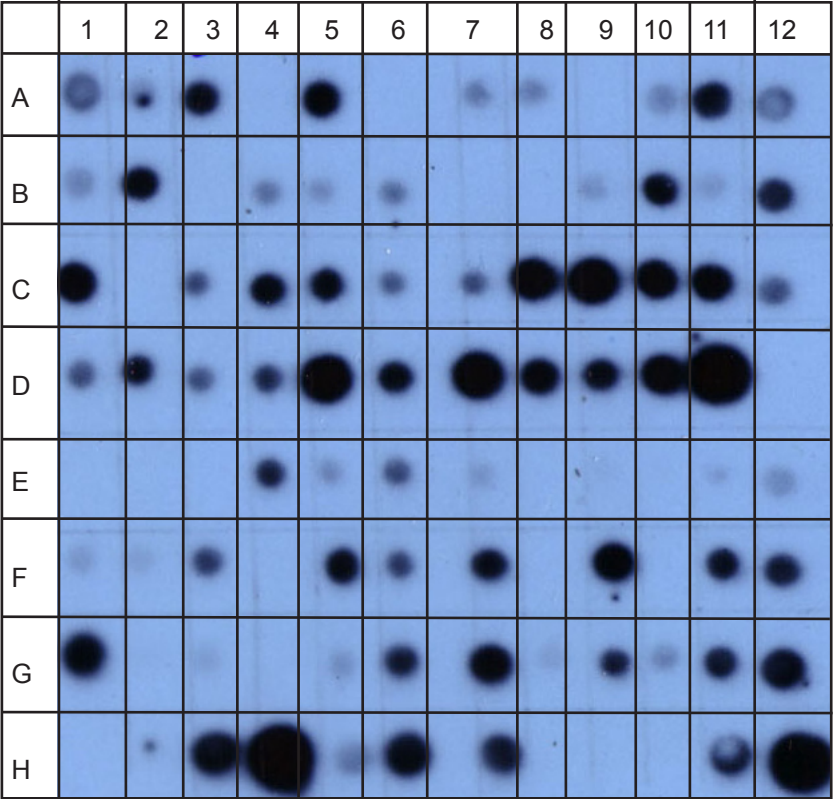

B = TESTER-SPECIFIC

Supplement: Additional file 9 — Differential screening of plate T-2 from T (tester) subtracted library was subjected to differential screening using driver-specific (A) and tester-specific (B) subtracted probes. [file 1471-2407-7-204-S9.pdf]

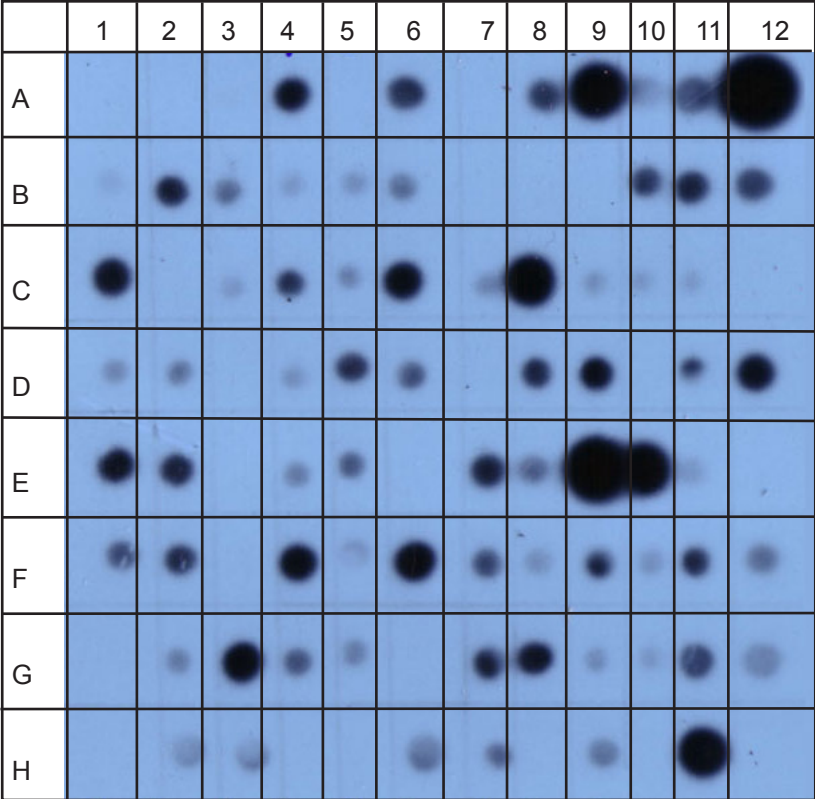

A = DRIVER-SPECIFIC

PLATE T-3

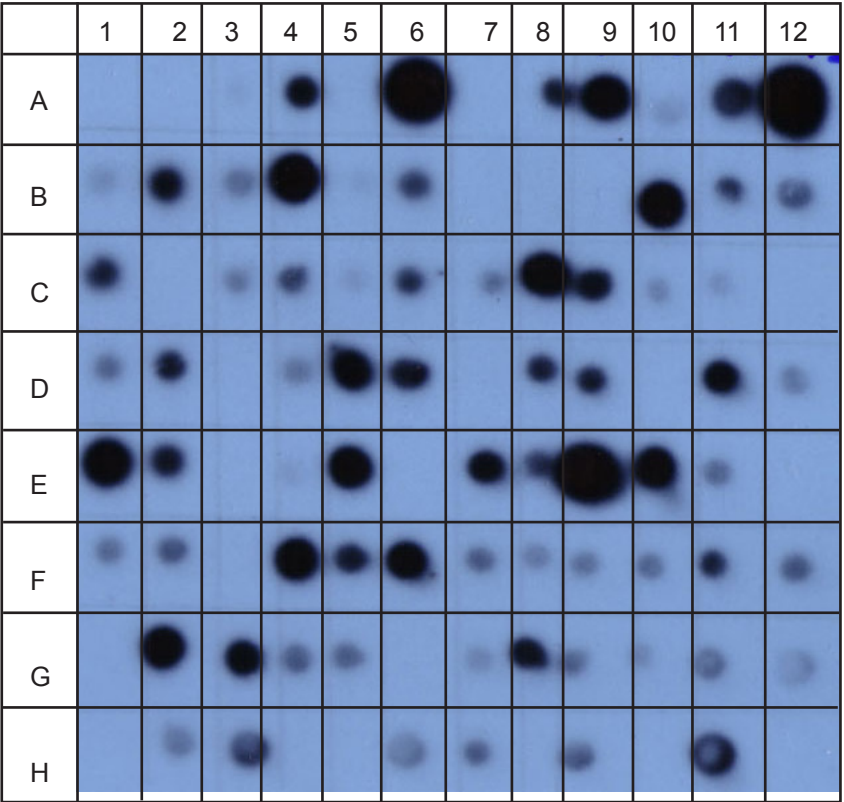

B = TESTER-SPECIFIC

Supplement: Additional file 10 — Differential screening of plate T-3 from T (tester) subtracted library was subjected to differential screening using driver-specific (A) and tester-specific (B) subtracted probes. [file 1471-2407-7-204-S10.pdf]

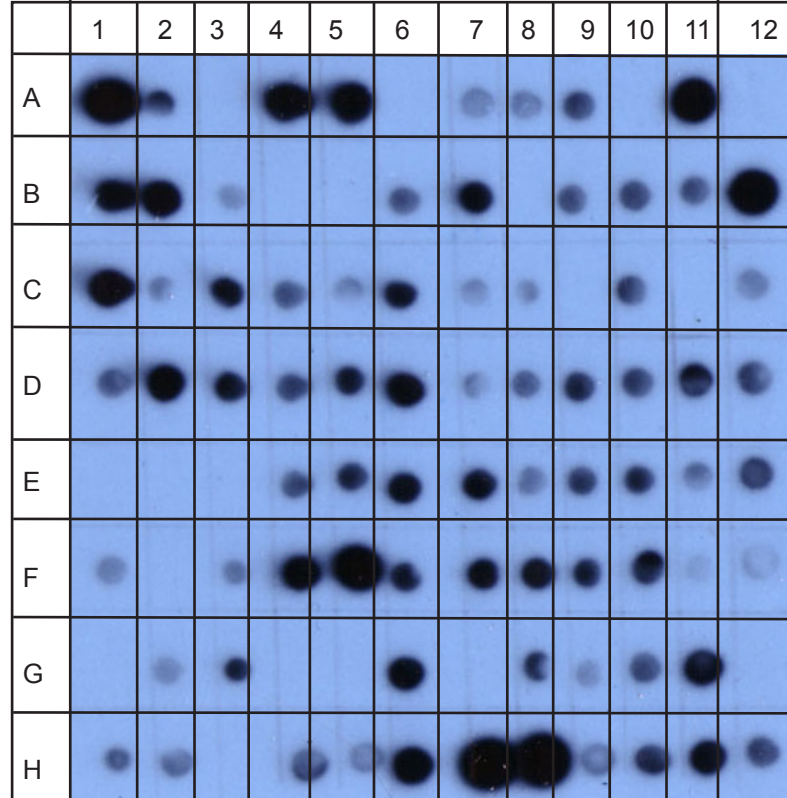

A = DRIVER-SPECIFIC

PLATE T-4

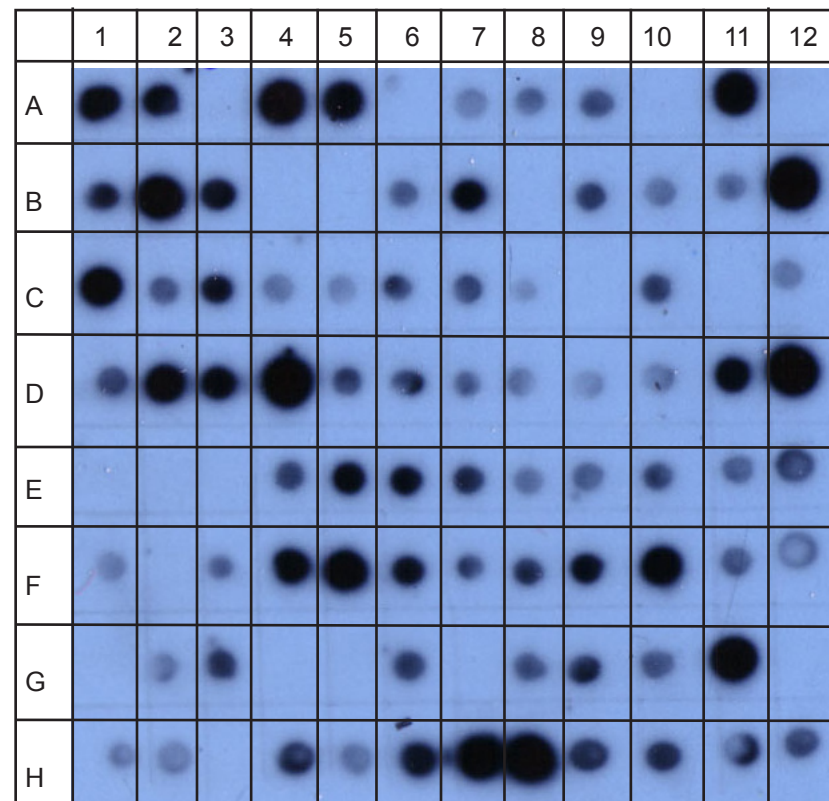

B = TESTER-SPECIFIC

Supplement: Additional file 11 — Differential screening of plate T-4 from T (tester) subtracted library was subjected to differential screening using driver-specific (A) and tester-specific (B) subtracted probes. [file 1471-2407-7-204-S11.pdf]

PLATE T-5

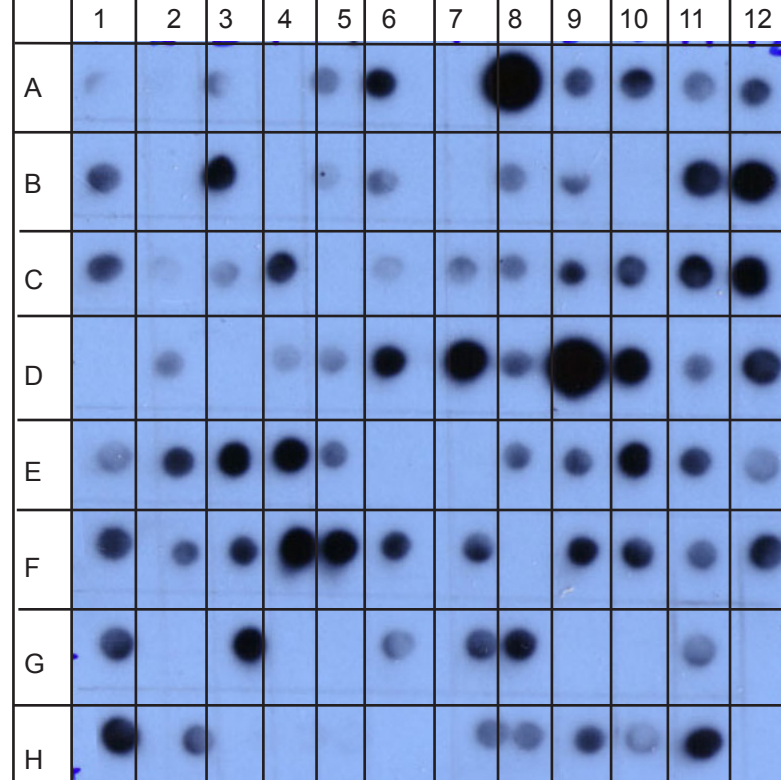

A = DRIVER-SPECIFIC

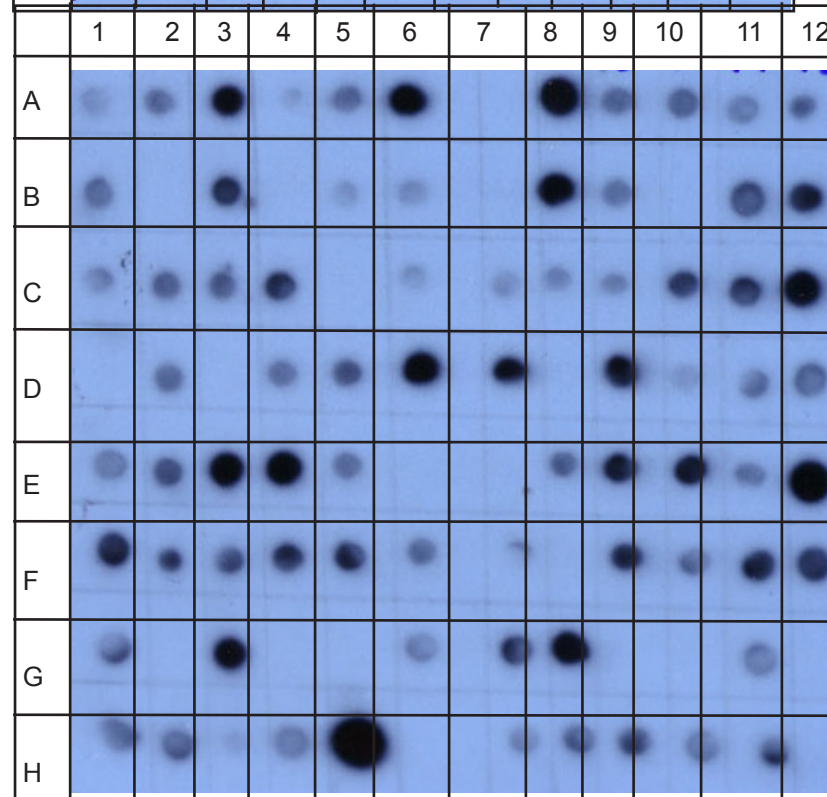

B = TESTER-SPECIFIC

Supplement: Additional file 12 — Differential screening of plate T-5 from T (tester) subtracted library was subjected to differential screening using driver-specific (A) and tester-specific (B) subtracted probes. [file 1471-2407-7-204-S12.pdf]

A = PLATE C-1

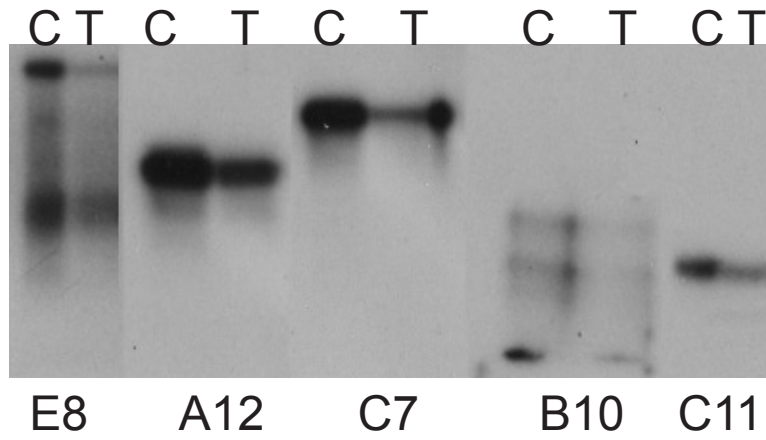

B = PLATE C-2

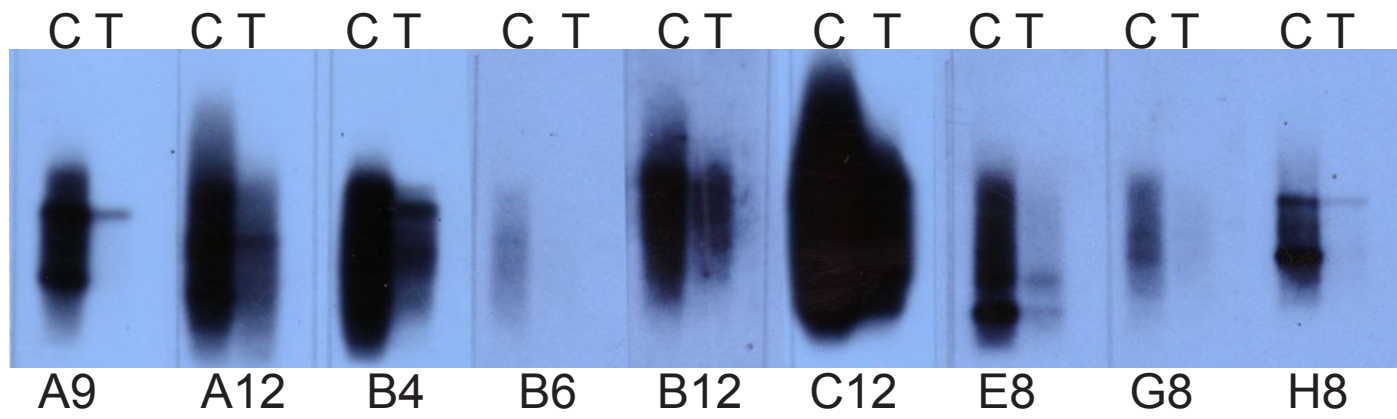

Supplement: Additional file 13 — Virtual Northern blot analysis of differential clones obtained from control bladder (C) subtracted library. A = Plate C-1 and B = Plate C-2 [file 1471-2407-7-204-S13.pdf]

A = PLATE T-1

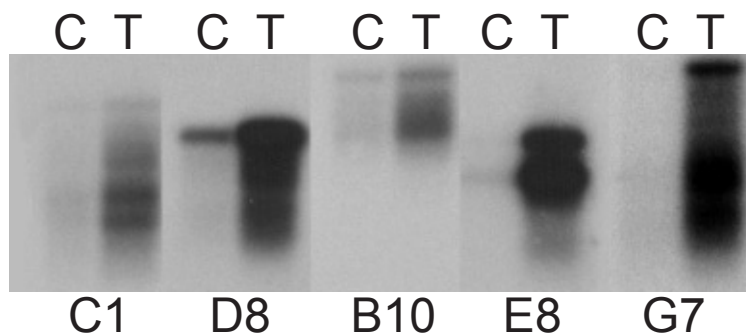

B = PLATE T-2

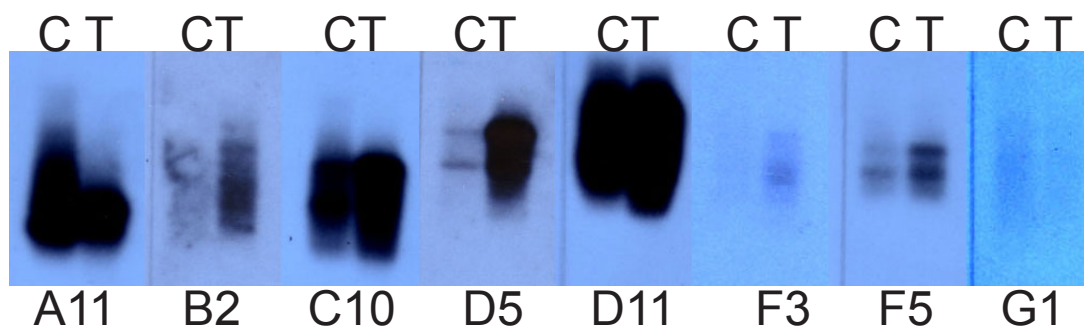

C = PLATE T-3

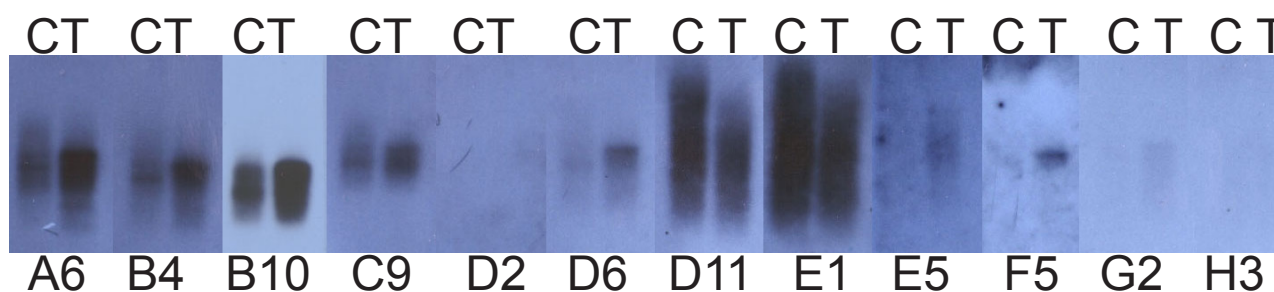

D = PLATE T-4

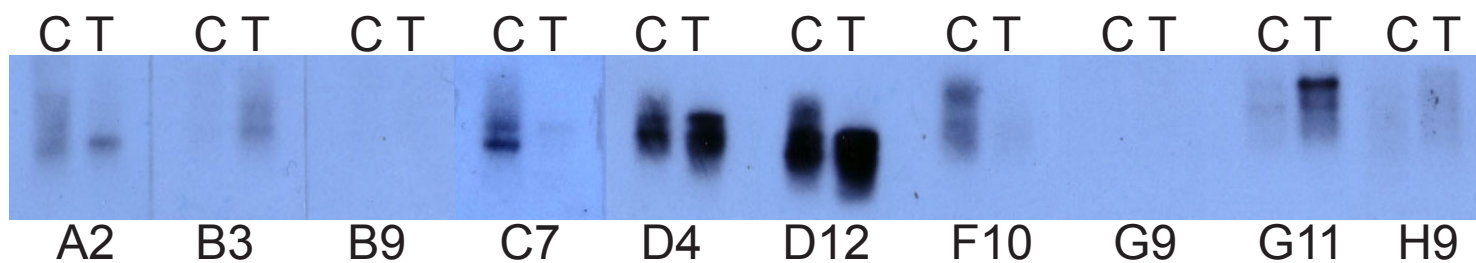

E = PLATE T-5

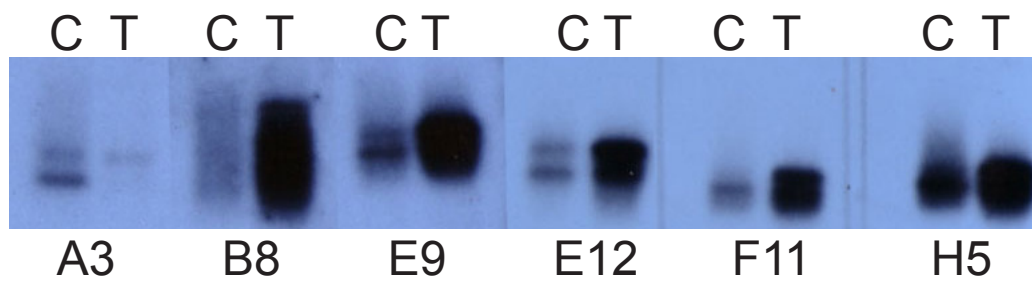

Supplement: Additional file 14 — Virtual Northern blot analysis of differential clones obtained from BCG-treated bladder (T) subtracted library. A = Plate T-1; B = Plate T-2; C = Plate T-3; D = Plate T-4; and E = Plate T-5. [file 1471-2407-7-204-S14.pdf]
