# Supplementary material for: Repeated BCG treatment of mouse bladder selectively stimulates small GTPases and HLA antigens and inhibits single-spanning uroplakins
Source: BMC Cancer. 2007 Nov 2;7:204. doi: 10.1186/1471-2407-7-204 (PMC2212656; doi:10.1186/1471-2407-7-204)
Supplement: Additional file 3 — Table 1 Oligonucleotides used [file 1471-2407-7-204-S3.pdf]

Table 1 **Oligonucleotides used**

SMART Oligo II A oligonucleotide	5’-AAGCAGTGGTATCAACGCAGAGTACGCrGrGrG-3’
SMART CDS primer II A	5’-AAGCAGTGGTATCAACGCAGAGTA-d(T)30-3’
SMART PCR primer II A	5’-AAGCAGTGGTATCAACGCAGAGT-3’
Adaptor 1	5’-CTAATACGACTCACTATAGGGCTCGAGCGGCCGCCCGGGCAGGT-3’<br>3’-GGCCCGTCCA-5’
PCR primer 1	5'-CTAATACGACTCACTATAGGGC-3'
Nested primer 1	5'-TCGAGCGGCCGCCCGGGCAGGT-3'
Adaptor 2R	5'-CTAATACGACTCACTATAGGGCAGCGTGGTCGCGGCCGAGGT-3'<br>3'-GCCGGCTCCA-5'
Nested primer 2R	5'-AGCGTGGTCGCGGCCGAGGT-3'
F1S plasmid primer	5'-AGTACGCTCAAGACGACAGAA-3'
R1S plasmid primer	5'-AAAGCAGTGGTAACAACGCAG-3'
