# Supplementary material for: Repeated BCG treatment of mouse bladder selectively stimulates small GTPases and HLA antigens and inhibits single-spanning uroplakins
Source: BMC Cancer. 2007 Nov 2;7:204. doi: 10.1186/1471-2407-7-204 (PMC2212656; doi:10.1186/1471-2407-7-204)
Supplement: Additional file 5 — Table 2. QOCR Primers [file 1471-2407-7-204-S5.pdf]

Table 2 **Q-PCR primers**

ABBREV	*Accession*	*Forward*	*Reverse*
Actb		tgataagtggccttggagtg	ctcagggcaggtgaaactgt
AQP3	NM_016689.2	gctctgcacgtctcctcttt	acatgcctgacaccactgaa
B2m	NM_009735	ctgggctggaacagagaaac	cctccctagacttcccaaca
C3	NM_009778	ctgctcaccctgcctagttc	acgatgagggctatgatgtg
CD9	NM_007657.3	ggcaaaggggaatctgtctt	gcttgtgaggtctggctctg
CTSS	AK150274	aacccttggctgtggaagag	ggatggctcacatggtttct
GBP2	XM_987479	tgtgccccaatgaaaaataa	cagaggaggaggtcggattc
GBP4	AK132266	tggggagggtgttgtttgta	ggtagggaccaggaggagag
GBP-5/Mpa2 like	NM_194336	tcagaagctggggaaaatgt	ggaaccatgaaggcaatgag
GSTA4	NM_010357	tcacggttgagccacctcta	cgtcgcagattacagcaaca
GSTM1/ GSTM5	NM_010358.4	ccatcacctctggcatgaat	gaacagccacctttccaaga
HLA-A/H2Aa	NM_010378	cattcgctggaactcaacaa	gaacccaaaggaagccaagt
H2-Bf	NM_008198	cagcctcttccctgtctcaa	GTAACTCGCCACctgttcat
HLA-DQA1/ H2-D1	AK159572	TTTCATGGATTGAGAATGCTT	AAGTTCCTTCATCTGCCATTT
HLA-E/ H2-T23	XM_00104712	GGTTTGGggttgtacttttgtt	TCTGGAAGGGATACGCTGAT
IIGP2	NM_019440	gctagaacggtggctttttg	acctcccaaactcaagacca
IIGP1	NM_021792	AATACCTGCCTCACGCTCAT	ACCTCGAAGACATCCCCTTT
LYZ	NM_017372.3	cacccagcacagcctactct	gatctggcaccctcttctgt
MMP7	AK144515	tccaggtatgcacggagaat	ttactggtgaggggagatgg
NKG7	NM_024253	AAGGACAGCAGGGTCATCAG	GTGGGAGGGACAGAGACAGA
Raig-2/GPRC5B*	NM_022420	CCCGTCACTTTCTTCTCTCC	TCCTGCatCAGCTTtCTcC
H2-A/HLA-DQB2/Rmcs1	AF015280	tgtcttgtgagggctgtttg	agtgtgctggaaacggaaat
TGTP	BC034256	CCAgGTAGGTCAAGGGTCTC	CGCTGGCTagtgAGTGAGAG
UBD	NM_023137	GGTCCCAAACCAAGGTCTCT	GCTCTTCATCACTGGGCTTC
UPK 1a	M_026815.2	gcccatcatcctctttttgt	cccaatttcatgtggcttct
UPK 1b	NM_178924.3	gctttctcctgtggctctgt	gccatcttgctgaccctaat
UPK 2	NM_175309.4	CCGAGTACAGAAGGGGACAT	tcggagcttagggaagtgtg
UPK 3a	NM_023478.1	gctcctatggtttcgcacat	gcccacttacatcccagttt
UPK 3b	NM_009476.2	gccactgaacttgtggttct	gcaatggggtcaagagtaag
Untr		TCAGGCATGAACCACCATAC	AACATCCACACGTCCAGTGA
