# Supplementary material for: Repeated BCG treatment of mouse bladder selectively stimulates small GTPases and HLA antigens and inhibits single-spanning uroplakins
Source: BMC Cancer. 2007 Nov 2;7:204. doi: 10.1186/1471-2407-7-204 (PMC2212656; doi:10.1186/1471-2407-7-204)
Supplement: Additional file 8 — Differential screening of plate T-1 from T (tester) subtracted library was subjected to differential screening using driver-specific (A) and tester-specific (B) subtracted probes. [file 1471-2407-7-204-S8.pdf]

PLATE T-1

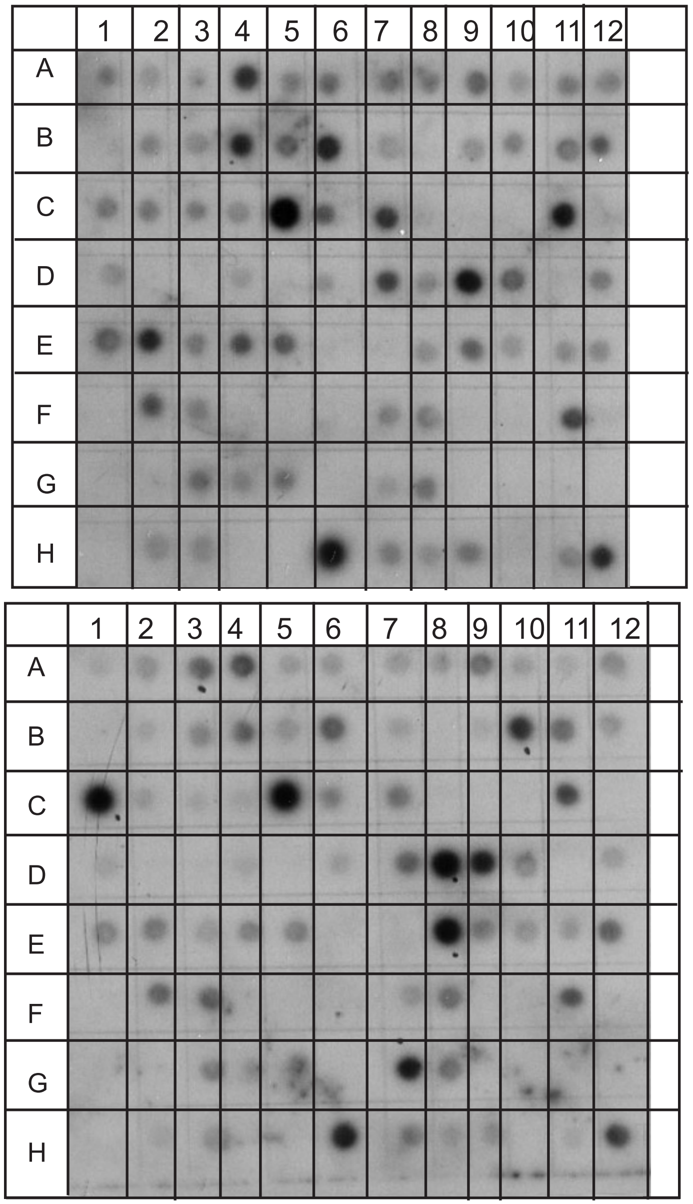

A = DRIVER-SPECIFIC

B = TESTER-SPECIFIC

Additional File 6.
