# Supplementary material for: Repeated BCG treatment of mouse bladder selectively stimulates small GTPases and HLA antigens and inhibits single-spanning uroplakins
Source: BMC Cancer. 2007 Nov 2;7:204. doi: 10.1186/1471-2407-7-204 (PMC2212656; doi:10.1186/1471-2407-7-204)
Supplement: Additional file 15 — Table 3. Genes up-regulated by BCG [file 1471-2407-7-204-S15.pdf]

Table 3 **Genes up-regulated by BCG**

Kown Genes

PlATE_CLONE	*NCBI*	*Link to UCSC Genome*	*Abbrev*	*Description*	*Location*	*Type*	*Function*
T2_F5, T5_H5, T5_F11	NM_009735	B2m	B2m	beta-2-microglobulin	Plasma Membrane	transmembrane receptor	
T2_B2	NM_009778	C3	C3	complement component 3	Extracellular Space	peptidase	Complement
T1_E12	AK098094	Cfb	CFB	complement factor B	Extracellular Space	peptidase	Complement
T5_E9 A	AK028366.1	Ctss	CTSS	Cathepsin S precursor (EC 3.4.22.27).	Cytoplasm	peptidase	
T5_B8	XM_987479	Gbp2	GBP2 (includes EG:14469)	guanylate nucleotide binding protein 2	Plasma Membrane	enzyme	GTPase activity
T1_F3	NM_008620	Gbp4	GBP4 (includes EG:17472)	guanylate nucleotide binding protein 4	Unknown	enzyme	GTPase activity
T1_A3	XM_001004461.1	Mpa21	GBP-5/Mpa2 like	macrophage activation 2 like	Unknown	other	GTPase activity
T1_D8, T4_G11, T5_E12, T5_M	AF015280.3	H2-Ab1	H2-A/HLA-DQB2/Rmcs1	histocompatibility 2 class II antigen A beta	Plasma Membrane	transmembrane receptor	Antigen
T2_C10, T3_B4, T3_B10	BC031711	H2-Aa	HLA-A/H2Aa	major histocompatibility complex, class I, A	Plasma Membrane	transmembrane receptor	Antigen
T3_C9	NM_010380	H2-D1	HLA-DQA1/ H2-D1	major histocompatibility complex, class II, DQ alpha 1	Plasma Membrane	transmembrane receptor	Antigen
T1_C11	AK150967	H2-K1	HLA-DQA1/H2-K1/ HLA-G	HLA-G histocompatibility antigen, class I, G	Plasma Membrane	transmembrane receptor	Antigen
T1_B10	XM_001004712	H2-T23	HLA-E/ H2-T23	major histocompatibility complex, class I, E	Plasma Membrane	transmembrane receptor	Antigen
T4_H9	BC103785.1	Igh-VJ558	IGH/ Igh-VJ558	immunoglobulin heavy chain complex	Unknown	other	Antigen processing
T1_G7	AK134155	Ighg	IGHG	Ig gamma-2B chain C region secreted form	Extracellular Space	other	Antigen binding
T3_E5, T4_B3	BC004649	Iigp1	IIGP1	interferon inducible GTPase 1	Unknown	enzyme	GTPase activity
T3_D2	AK142291	Iigp2	IIGP2	interferon inducible GTPase 2	Cytoplasm	enzyme	GTPase activity
T2_D11, T3_A6, T4_D4	AC139638.14	Lyzs	LYZ	lysozyme	Extracellular Space	enzyme	
T2_F3	NM_010810	Mmp7	MMP7	matrix metallopeptidase 7 (matrilysin, uterine)	Extracellular Space	peptidase	
T3_F5	NM_024253	Nkg7	NKG7	natural killer cell group 7 sequence	Plasma Membrane	other	
T2_A11	NM_022420	Gprc5b	Raig-2/GPRC5B*	Retinoic acid-induced gene 2 protein/ GPCR5B	Plasma Membrane	G-protein coupled receptor	Retinoic acid-GPCR interaction
T2_D5	AK172580	Tgtp	TGTP	T-cell specific GTPase	Unknown	enzyme	GTPase activity
T1_E8	BC027627	Ubd	UBD	ubiquitin D	Nucleus	other	

Unkown Genes

PlATE_CLONE	*NCBI*	*Link to UCSC Genome*	*Abbrev*	*Description*	*Location*	*Type*	*Function*
T4_A2, T4_M13	XM_984682.1	EF660528	AW112010/ EF660528	small secreted protein interferon-induced	Unknown	Unknown	
