# Supplementary material for: Repeated BCG treatment of mouse bladder selectively stimulates small GTPases and HLA antigens and inhibits single-spanning uroplakins
Source: BMC Cancer. 2007 Nov 2;7:204. doi: 10.1186/1471-2407-7-204 (PMC2212656; doi:10.1186/1471-2407-7-204)
Supplement: Additional file 16 — Table 4. Genes down-regulated by BCG. [file 1471-2407-7-204-S16.pdf]

Table 4 **Genes Downregulated by BCG**

Known Genes

Plate_Clone	*NCBI*	*Link to UCSC Genome*	*Abbrev*	*Description*	*Location*	*Type*	*Function*
C1_E8	AL837521.6	Aqp3	AQP3	aquaporin 3	Plasma Membrane	transporter	
C2_H8	NM_007657.3	Cd9	CD9	CD9 antigen	Plasma Membrane	other	protein binding
C2_B12	NM_025902.2	Cisd2	Cisd2/ZCD2	CDGSH iron sulfur domain 2	Unknown	other	
C2_A12	NM_010357.2	Gsta4	GSTA4	glutathione S-transferase, alpha 4	Cytoplasm	enzyme	glutathione transferase activity
C1_C7, C2_C12, C2_M13	BC046758.1	Gstm1	GSTM1/ GSTM5	glutathione S-transferase, mu 1,	Cytoplasm	enzyme	glutathione transferase activity
C2_E8	AC158993.2	Lgi4	LGI4	leucine-rich repeat LGI family member 4	Extracellular Space	other	
C1_D1	NM_025348.1	Ndufa3	NDUFA3	NADH dehydrogenase (ubiquinone) 1 alpha subc	Cytoplasm	enzyme	oxireductase activity
C1_C11	NM_023133.1	Rps19	RPS19	ribosomal protein S19	Cytoplasm	other	
C1_A12	XM_001000192.1	Sprr2a	SPRR2G	small proline-rich protein 2A	Cytoplasm	other	
C2_A9	AL772308.4	Upk3a	UPK3A	uroplakin 3A	Plasma Membrane	other	

Unknown Genes

Plate_Clone	*NCBI*	*Link to UCSC Genome*	*Abbrev*	*Description*	*Location*	*Type*	*Function*
C2_G8	AC161870.4		clone RP24-70M22	BAC clone RP24-70M22 from chromosome 14	Unknown	Unknown	
C1_B10, C1_D6, C1_E4, C1_G2, C1_H7	AY999076.1		mitochondrion	Mus musculus cell-line NIH/3T3 mitochondrion	Unknown	Unknown	
C1_E11	BC012020.1		clone IMAGE:4500967	Hypothetical protein	Unknown	Unknown	
C2_B4	EF108344.1		mitochondrion	Mus musculus domesticus strain WSB/EiJ	Unknown	Unknown	
C2_B6	NM_025856.2	1700029G01Rik	1700029G01RIK	preimplantation mouse development	Unknown	Unknown	
C1_E12	AK153940.1		A130040M12RIK	RIKEN cDNA A130040M12 gene	Unknown	Unknown	
