# Supplementary material for: Repeated BCG treatment of mouse bladder selectively stimulates small GTPases and HLA antigens and inhibits single-spanning uroplakins
Source: BMC Cancer. 2007 Nov 2;7:204. doi: 10.1186/1471-2407-7-204 (PMC2212656; doi:10.1186/1471-2407-7-204)
Supplement: Additional file 17 — Table 5. Ingenuity Summary BCG. [file 1471-2407-7-204-S17.pdf]

Table 5. BCG-specific Summary IPA

TOP NETWORKS	ASSOCIATED NETWORKS FUNCTIONS						Score
1	Cell Signaling, Immune Response, Connective Tissue Disorders						45
2	Cancer, Cardiac Hyplerplasia/Hyperproliferation, Cardiovascular Disease						3
DISEASES AND DISORDERS		Lower p value	Lower .-log (p-value)	Upper p value	Upper .-log (p-value)	Threshold . -log (p-value)	#Molecules
	Connective Tissue Disorders	2.98E-07	6.53	2.99E-02	1.52	1.35	6
	Immunological Disease	5.40E-07	6.27	3.08E-02	1.51	1.35	7
	Inflammatory Disease	2.10E-06	5.68	2.80E-02	1.55	1.35	8
	Skeletal and Muscular Disorders	2.10E-06	5.68	2.60E-02	1.59	1.35	5
	Neurological Disease	2.56E-06	5.59	1.20E-02	1.92	1.35	5
MOLECULAR AND CELLULAR FUNCTIONS		Lower p value	Lower .-log (p-value)	Upper p value	Upper .-log (p-value)	Threshold . -log (p-value)	#Molecules
	Cell Signaling	8.82E-13	12.05	1.20E-03	2.92	1.35	12
	Cellular Growth and Proliferation	5.34E-06	5.27	2.80E-02	1.55	1.35	6
	Cell-To-Cell Signaling and Interaction	2.66E-05	4.58	3.10E-02	1.51	1.35	7
	Cell Death	3.97E-05	4.40	3.00E-02	1.52	1.35	5
	Cellular Movement	8.94E-04	3.05	3.00E-04	3.52	1.35	5
PHYSIOLOGYCAL SYSTEM DEVELOPMENT AND FUNCTION		Lower p value	Lower .-log (p-value)	Upper p value	Upper .-log (p-value)	Threshold . -log (p-value)	#Molecules
	Immune Response	8.82E-13	12.05	3.10E-02	1.51	1.35	12
	Hematological System Development and Function	5.34E-06	5.27	3.10E-02	1.51	1.35	6
	Immune and Lymphatic System Development	1.35E-04	3.87	3.10E-02	1.51	1.35	7
	Tissue Morphology	3.60E-04	3.44	1.40E-02	1.85	1.35	5
	Skeletal and Muscle System Development	8.23E-04	3.08	8.70E-02	1.06	1.35	5
TOP CANONICAL PATHWAYS					p value	.-log (p-value)	Ratio
	Antigen Presentation				5.27E-13	12.28	6/39 (0.154)
	Protein Ubiquitination				7.72E-04	3.11	3/200 (0.015)
	Complement and Coagulation Cascades				2.10E-03	2.68	2/70 (0.029)
	Wnt/beta-catenin				7.10E-01	0.15	2/162 (0.012)
	Cell Cycle: G2/M Dna Damage Checkpoint Regulation				3.65E-02	1.44	1/142 (0.024)
