# Supplementary material for: Repeated BCG treatment of mouse bladder selectively stimulates small GTPases and HLA antigens and inhibits single-spanning uroplakins
Source: BMC Cancer. 2007 Nov 2;7:204. doi: 10.1186/1471-2407-7-204 (PMC2212656; doi:10.1186/1471-2407-7-204)
Supplement: Additional file 18 — Table 6. Ingenuity Summary Control. [file 1471-2407-7-204-S18.pdf]

Table 6 **IPA analysis of Control-specific genes**

TOP NETWORKS	*ASSOCIATED NETWORKS FUNCTIONS*					*Score*
1	Cancer, Cellular Movement, Cell-To-Cell Signaling					10
2	Vitamin and Mineral Metabolism, Carbohydrate metabolism, Cell Signalling					3
3	Cancer, Cell Morphology, Cell-To-Cell Signaling					3
4	Cellular Development, Immunological Disease, Cell Cycle					3

DISEASES AND DISORDERS	*Lower* p value	.-log (p-value)	*Upper* p value	.-log (p-value)	*Threshold* . -log (p-value)	*#Molecules*
Genetic Disorder	1.17E-03	2.93	1.10E-02	1.96	1.35	1
Hematological Disease	1.17E-03	2.93	9.30E-02	1.03	1.35	2
Immunological Disease	1.17E-03	2.93	3.50E-03	2.46	1.35	1
Renal and Urological Disease	1.17E-03	2.93	1.40E-02	1.85	1.35	2
Skeletal and Muscular Disorders	2.34E-03	2.63	8.10E-03	2.09	1.35	1

MOLECULAR AND CELLULAR FUNCTIONS	*Lower* p value	.-log (p-value)	*Upper* p value	.-log (p-value)	*Threshold* . -log (p-value)	*#Molecules*
Cell Morphology	5.86E-04	3.23	3.40E-02	1.47	1.35	3
Amino Acid Metabolism	1.11E-03	2.95	1.10E-04	3.96	1.35	2
Cell Death	1.17E-03	2.93	1.90E-03	2.72	1.35	2
Cell-To-Cell Signaling	1.17E-03	2.93	3.80E-02	1.42	1.35	1
Cellular Assembly and Organization	1.17E-03	2.93	2.60E-02	1.59	1.35	1

PHYSIOLOGYCAL SYSTEM DEVELOPMENT AND FUNCTION	*Lower* p value	.-log (p-value)	*Upper* p value	.-log (p-value)	*Threshold* . -log (p-value)	*#Molecules*
Nervous System and Development and Function	5.86E-04	3.23	3.60E-03	2.44	1.35	2
Reproductive System Development and Function	5.86E-04	3.23	4.60E-02	1.34	1.35	3
Tissue Morphology	5.86E-04	3.23	3.90E-02	1.41	1.35	3
Hematological System Development and Function	1.17E-03	2.93	4.70E-02	1.33	1.35	2
Immune Response	1.17E-03	2.93	4.70E-02	1.33	1.35	2

TOP CANONICAL PATHWAYS	p value	.-log (p-value)	Ratio
Glutathione Metabolism	1.11E-03	2.95	2/102 (0.2)
NRF2-mediated Oxidative Stress	2.58E-03	2.59	2/142 (0.014)
Aryl Hydrocarbon Receptor Signalling	2.58E-03	2.59	2/147 (0.014)
LPS/IL-1 Mediated Inhibition of RXR Function	4.07E-03	2.39	2/170 (0.012)
Metabolism of Xenobiotics by Cytochrome P450	6.46E-03	2.19	2/213 (0.009)

Top Tox List	p value	.-log (p-value)	Ratio
Oxidative Stress	4.79E-04	3.32	2/57 (0.035)
Xenobiotic Metabolism	2.42E-03	2.62	2/129 (0.016)
Mitochondrial Dysfuction	4.37E-02	1.36	1/76 (0.013)
